# Deep-learning-based generation of synthetic 6-minute MRI from 2-minute MRI for use in head and neck cancer radiotherapy

**DOI:** 10.3389/fonc.2022.975902

**Published:** 2022-11-08

**Authors:** Kareem A. Wahid, Jiaofeng Xu, Dina El-Habashy, Yomna Khamis, Moamen Abobakr, Brigid McDonald, Nicolette O’ Connell, Daniel Thill, Sara Ahmed, Christina Setareh Sharafi, Kathryn Preston, Travis C. Salzillo, Abdallah S. R. Mohamed, Renjie He, Nathan Cho, John Christodouleas, Clifton D. Fuller, Mohamed A. Naser

**Affiliations:** ^1^ Department of Radiation Oncology, The University of Texas MD Anderson Cancer Center, Houston, TX, United States; ^2^ Elekta AB, Stockholm, Sweden; ^3^ Department of Clinical Oncology and Nuclear Medicine, Menoufia University, Shebin Elkom, Egypt; ^4^ Department of Clinical Oncology and Nuclear Medicine, Faculty of Medicine, Alexandria University, Alexandria, Egypt

**Keywords:** deep learning, MRI, head and neck cancer, adaptive radiotherapy, generative adveserial network

## Abstract

**Background:**

Quick magnetic resonance imaging (MRI) scans with low contrast-to-noise ratio are typically acquired for daily MRI-guided radiotherapy setup. However, for patients with head and neck (HN) cancer, these images are often insufficient for discriminating target volumes and organs at risk (OARs). In this study, we investigated a deep learning (DL) approach to generate high-quality synthetic images from low-quality images.

**Methods:**

We used 108 unique HN image sets of paired 2-minute T2-weighted scans (2mMRI) and 6-minute T2-weighted scans (6mMRI). 90 image sets (~20,000 slices) were used to train a 2-dimensional generative adversarial DL model that utilized 2mMRI as input and 6mMRI as output. Eighteen image sets were used to test model performance. Similarity metrics, including the mean squared error (MSE), structural similarity index (SSIM), and peak signal-to-noise ratio (PSNR) were calculated between normalized synthetic 6mMRI and ground-truth 6mMRI for all test cases. In addition, a previously trained OAR DL auto-segmentation model was used to segment the right parotid gland, left parotid gland, and mandible on all test case images. Dice similarity coefficients (DSC) were calculated between 2mMRI and either ground-truth 6mMRI or synthetic 6mMRI for each OAR; two one-sided t-tests were applied between the ground-truth and synthetic 6mMRI to determine equivalence. Finally, a visual Turing test using paired ground-truth and synthetic 6mMRI was performed using three clinician observers; the percentage of images that were correctly identified was compared to random chance using proportion equivalence tests.

**Results:**

The median similarity metrics across the whole images were 0.19, 0.93, and 33.14 for MSE, SSIM, and PSNR, respectively. The median of DSCs comparing ground-truth vs. synthetic 6mMRI auto-segmented OARs were 0.86 vs. 0.85, 0.84 vs. 0.84, and 0.82 vs. 0.85 for the right parotid gland, left parotid gland, and mandible, respectively (equivalence p<0.05 for all OARs). The percent of images correctly identified was equivalent to chance (p<0.05 for all observers).

**Conclusions:**

Using 2mMRI inputs, we demonstrate that DL-generated synthetic 6mMRI outputs have high similarity to ground-truth 6mMRI, but further improvements can be made. Our study facilitates the clinical incorporation of synthetic MRI in MRI-guided radiotherapy.

## Introduction

Head and neck cancer (HNC) is among the most common malignancies globally (1). A core treatment modality for HNC patients is radiotherapy (RT) (2). The current clinical standard for HNC RT planning involves pre-therapy imaging using computed tomography (CT). However, adaptive RT (ART) using magnetic resonance imaging (MRI)-guided approaches offers distinct advantages over the current clinical standard, such as increased soft-tissue contrast and radiation-free intra-treatment imaging, which can be leveraged for improved tumor control and decreased side effects (3, 4). Therefore, it is predicted that MRI-guided ART will play an increasingly important role in HNC patient management.

Anatomical MRI sequences, particularly T2-weighted (T2w) images, are routinely acquired during online and offline MRI-guided ART and may be used for segmentation of target structures, i.e., primary tumors and metastatic lymph nodes, and organs at risk (OARs) (5). Specifically, in the Elekta Unity MR-linac HNC workflow, quick T2w images with low contrast-to-noise ratio, typically acquired over 2 minutes, are often used for on-board setup imaging to minimize treatment times. However, these quick setup images are not always sufficient for the optimal discrimination of target structures and OARs, especially when deciding if adaptive re-planning is necessary (5). Longer scan times, typically performed over 6 minutes, can be employed to improve image contrast-to-noise ratio and thus overall image quality, but routine use must be balanced against the patient’s comfort and the treatment schedule. Therefore, the rapid acquisition of high-quality T2w scans is an unmet need in MRI-guided ART workflows.

Deep learning (DL) has found wide success in a variety of domains for RT-related medical imaging applications such as target and OAR segmentation (6–11) and outcome prediction (12, 13). One less routinely studied domain is synthetic image generation, i.e., mapping an input image to an output image. Recent work has highlighted the utility of DL for synthetically generating CT images from MRI sequences (14–21), MRI sequences from CT images (22–26), and MRI sequences from other MRI sequences (27–31). However, to date, no studies have investigated the feasibility of using DL to generate high-quality synthetic MRI sequences from low-quality MRI sequences to decrease the required scan time for HNC-related imaging.

**Figure 1:**
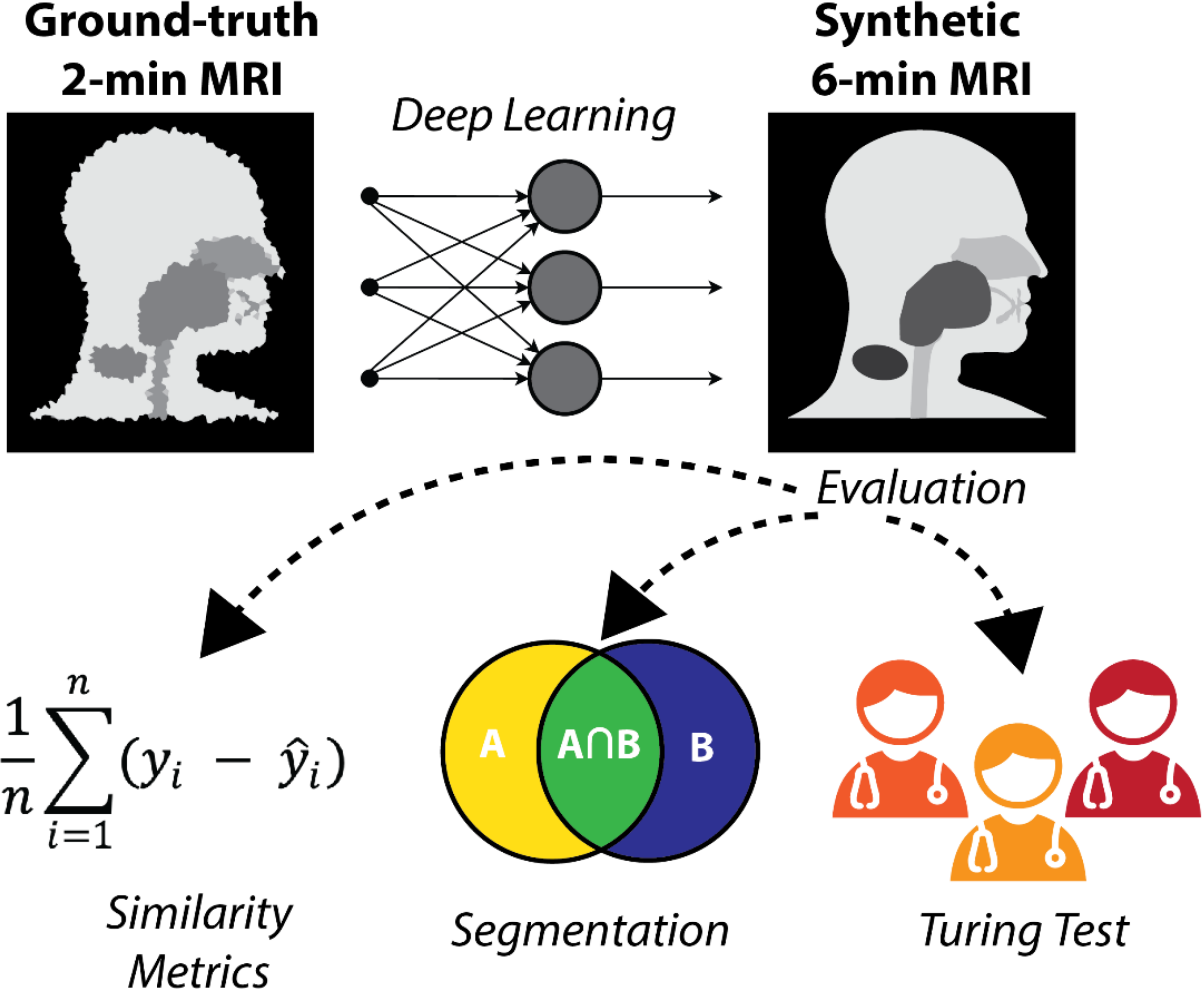
Study overview. 2-minute MRI scans are used as input to a deep learning model to generate synthetic 6-minute MRI scans. The synthetic 6-minute scans are compared to ground-truth 6-minute scans through various quantitative and qualitative methods.

In this study, we evaluated the feasibility of generating synthetic 6-minute T2w MRI sequences from 2-minute T2w MRI sequences for use in MRI-guided RT workflows. Using paired 2-minute and 6-minute scans, we trained a DL network to generate high-quality synthetic 6-minute scans. We employed various quantitative and qualitative evaluation techniques, including a clinician-based visual Turing test, to demonstrate the potential acceptability of synthetic image generation for use in MRI-guided RT workflows. An overview of the study is shown in **Figure 1**.

## Methods

### Imaging data

Data were retrospectively collected from a clinical trial investigating MRI-guided ART (National Clinical Trial Identifier: NCT04075305) and an internal volunteer imaging study under HIPAA-compliant protocols approved by The University of Texas MD Anderson Cancer Center’s Institutional Review Board (PA18-0341, PA14-1002, RCR03-0800). Paired 2-minute T2w MRI scans and 6-minute T2w MRI scans, acquired during the same imaging session, were collected for 53 participants (50 HNC patients; three volunteers). These MRI sequences have been established for use with the Unity MR-linac HNC workflow (5). Details of the participants’ demographic and clinical characteristics are shown in **Appendix A**. For a subset of 10 HNC patients, multiple intra-RT paired image sets were available; the total number of paired image sets varied from 2-9 for each patient (total number of paired image sets = 56). One HNC patient had nine intra-RT 2-minute scans and one pre-RT 6-minute scan available, so a deformable image registration in ADMIRE v. 3.42 (Elekta AB, Stockholm, Sweden) was performed between the 6-minute and 2-minute scans to yield nine image sets. One volunteer had two additional paired image sets available. The remaining 21 participants (19 HNC patients pre-RT; two volunteers) had one paired image set available. In total, 108 unique paired image sets were available for use. All participants were scanned on the same scanner, a 1.5 T Elekta Unity MR-linac device. The acquisition characteristics of the 2-minute and 6-minute scans are shown in **Table 1**. Both the 2-minute and 6-minute scans were 3D Turbo spin echo acquisitions. Additional details on these MRI acquisitions can be found in literature by McDonald et al. (5). All participants were immobilized with a thermoplastic mask, which minimized differences in anatomical positioning between sequence acquisitions. Notably, the 2-minute scans had a slightly longer field of view superiorly and inferiorly compared to the 6-minute scans. All imaging data were collected in Digital Imaging and Communications in Medicine (DICOM) format.

**Table 1:** MRI sequence acquisition parameters for the 2-minute and 6-minute MRI scans used in this study.

Acquisition Parameter	2-minute	6-minute
Repetition time (ms)	1535	2100
Echo time (ms)	278	375
Echo train length	114	150
Flip angle (°)	90	90
Slice thickness (mm)	2.0	2.2
In-plane resolution (mm)	0.83	0.68
Slice gap (mm)	1.0	1.1
Acquisition matrix	268x268	432x433
Pixel bandwidth (Hz/px)	740	459
Number of averages	1	2
Number of axial slices	300	227

### Data partitioning

For the purposes of model training and evaluation, we split data into separate training and test sets. The 11 HNC patients with multiple image sets, three volunteer cases, and a random sample of 21 HNC patients with single image sets were included in the training set, leading to a total of 90 unique paired image sets, i.e., ~20,000 slices, for model training. The remaining 18 HNC patients with single image sets were used for the test set, leading to a total of 18 unique paired image sets for model testing.

### Deep learning network

**Figure 2:**
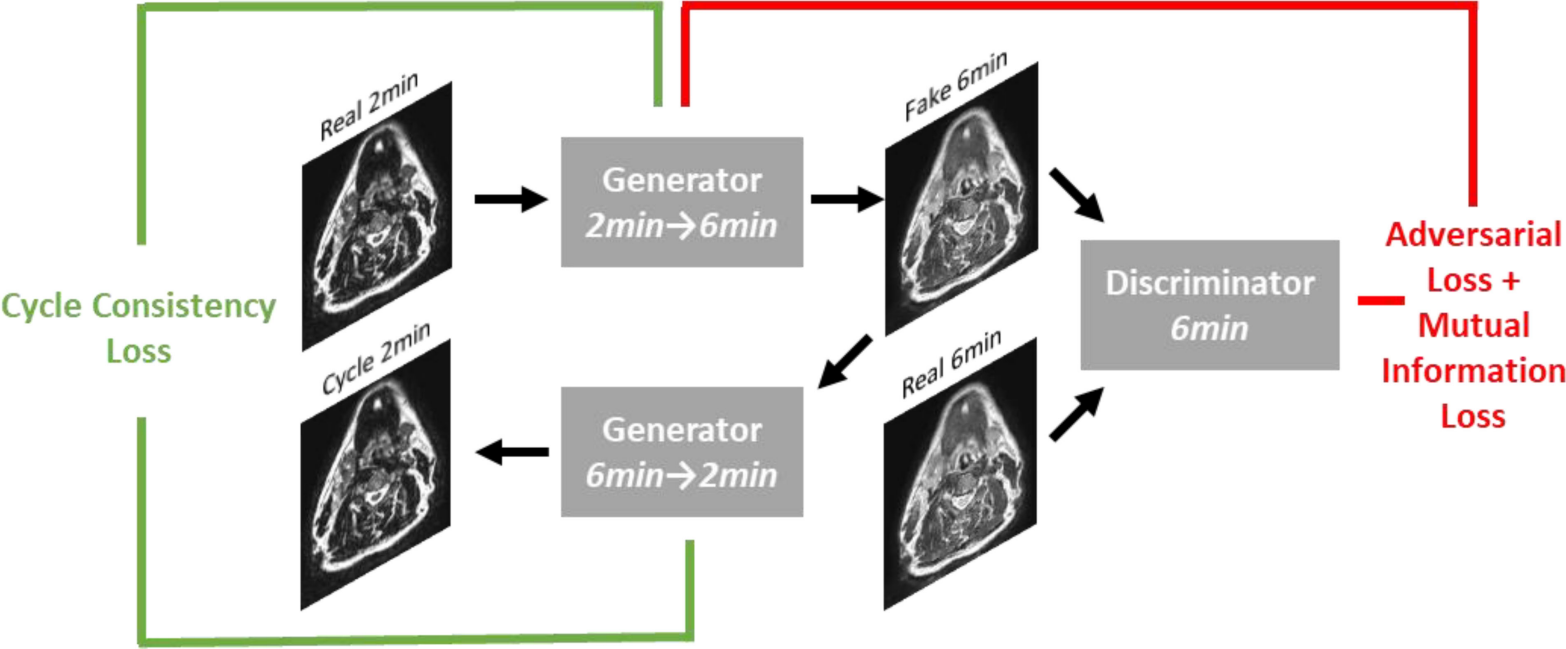
Overview of loss functions used in deep learning network. For simplicity, only the first half of the CycleGAN is shown where a fake 6-minute scan is generated from a real 2-minute scan. An analogous process occurs to generate a fake 2-minute scan from a real 6-minute scan.

A DL generative adversarial neural network (GAN) (32) model based on the CycleGAN architecture (33) using paired T2w 2-minute and T2w 6-minute scans was implemented in Tensorflow (34). Specifically, our model implementation draws inspiration from work performed by Johnson et al. (35), which showed promising results for image translation. The overall structure of the generative networks is based on the classic 2D Resnet encoder-decoder structure and a PatchGAN discriminator network (36–38); inputs and outputs to the model were of size 512x512. Additional technical details on the DL architecture can be found in **Appendix B**. To obtain better quantitative synthetic results, we adopted an additional mutual information loss in addition to the original adversarial and cycle-consistency losses. The mutual information term (39) was calculated between the synthetic and the ground-truth images, and the negative mutual information was minimized. The weight parameters for the adversarial loss, cycle-consistency loss, and mutual information loss were set to 1, 10, and 1, respectively. The learning rate was fixed as 2e-4 for the first half of all training epochs and was linearly decayed for the second half of training; 200 total training epochs were used. **Figure 2** shows an overview of the DL loss functions.

### Deep learning data processing

Several simple data processing steps were performed prior to training the deep learning model. First, 6-minute T2w images were rigidly registered with corresponding 2-minute T2w images. Previous studies have noted the importance of intensity standardization in DL-based MRI image synthesis (40); therefore, we also applied these steps to our processing pipeline. Specifically, an N4 bias field correction (41) was applied to remove additional low-frequency non-uniform artifacts. In addition, a z-score normalization, where the mean and standard deviation were calculated from the voxel values in the range of [0.25% 99.75%], was applied to 2-minute T2w images, followed by an additional rescaling to a range of [-1.0, 1.0]. The registered 6-minute T2w images were also rescaled to a range of [-1.0, 1.0]. The final trained DL model was implemented in ADMIRE v. 3.42 (Elekta AB, Stockholm, Sweden) and subsequently applied to the 2-minute test case scans to generate the synthetic 6-minute images for data analyses. Each synthetic image took approximately 1 second to generate on a NVIDIA V100 GPU, with an additional approximate 30 seconds after applying the N4 bias field correction. All DL inputs and outputs for the test set cases are made publicly available on Figshare: 10.6084/m9.figshare.20099252.

### Data analyses

All analyses were performed in Python v. 3.9.7 (42). DICOM images and RT structure files were converted to Neuroimaging Informatics Technology Initiative format using the DICOMRTTool Python package (43). Subsequently, all processing operations were performed using Numpy (44) arrays. All analysis code is available on GitHub: https://github.com/kwahid/2min_6min_synthetic_MRI.

### Image similarity evaluation

To quantitatively compare image similarity between ground-truth 6-minute scans and synthetic 6-minute scans, we used several commonly implemented metrics for image similarity. Specifically, we implemented the mean squared error (MSE), structural similarity index (SSIM), and the peak signal-to-noise ratio (PSNR). The metrics were chosen because of their widespread ubiquity in contemporary literature. All metrics were derived from the scikit-image Python package (45). SSIM was calculated using a window size of 11 as recommended in literature (46), and the PSNR was calculated using a data range based on the maximum and minimum value of the ground-truth image. Image similarity metrics were used to compare images globally (whole image) and at a region of interest (ROI) level. ROIs were based on the auto-segmented OAR structures on 2-minute scans described below (left parotid gland, right parotid gland, left submandibular gland, right submandibular gland, mandible, spinal cord, and brainstem) and an external mask of the head/neck region generated by the Otsu method (47). Bounding boxes were created around each ROI to perform the similarity calculation. Before comparison, synthetic images were resampled to the same image space as the ground-truth images using an order-3 B-spline interpolator. In addition, ground-truth images were N4 bias field corrected to ensure a fair comparison with the N4 bias field corrected synthetic images; N4 bias field post-processing was performed in ADMIRE v. 3.42 (Elekta AB, Stockholm, Sweden). Additionally, both images were z-score normalized before metric evaluation to ensure that the analysis was independent of the scale of intensity values, as suggested in previous literature (48), therefore MSE and PSNR calculations can be interpreted as a normalized MSE and PSNR.

### Auto-segmentation evaluation

In **Appendix C**, we show that clinicians prefer 6-minute scans over 2-minute scans for OAR visualization. As a proxy for a human segmentation task, we implemented auto-segmentation of common HNC OARs. A previously developed DL HNC OAR auto-segmentation model available in ADMIRE v. 3.42 (Elekta AB, Stockholm, Sweden) that was trained on 2-minute T2w scans was used for the analysis. The model had previously shown superior performance to gold-standard segmentations and therefore was trusted as a reasonable proxy for clinician-generated segmentations. Additional details of the auto-segmentation model can be found in McDonald et al. (49). The left parotid gland, right parotid gland, left submandibular gland, right submandibular gland, mandible, spinal cord, and brainstem were auto-segmented for all test patients on 2-minute, ground-truth 6-minute, and synthetic 6-minute scans using the pre-trained model (representative examples shown in **Appendix D**). Dice similarity coefficient (DSC) and average surface distance (ASD) values were calculated between 2-minute scans and either ground-truth 6-minute scans or synthetic 6-minute scans for each OAR. DSC and ASD were selected because of their general ubiquity in auto-segmentation studies and ability to discriminate volumetric and surface-level segmentation quality, respectively (50). Before comparison, OAR masks were resampled to the same image space as the ground-truth 6-minute image using a nearest neighbor interpolator. All auto-segmentation metrics were calculated using the surface-distances Python package (51). Only OARs with metric values better than previously reported interobserver variability (IOV) values derived from 2-minute images, as determined from McDonald et al. (49), were used for further analysis (additional details in **Appendix D**). Paired two one-sided t-tests (TOST) (52) were applied between metric values of the ground-truth 6-minute scans and synthetic 6-minute scans for each OAR to determine equivalence; p-values less than 0.05 were considered statistically significant. The logic behind this analysis is that resultant auto-segmented OARs on ground-truth and synthetic 6-minute scans should be similar, i.e., statistically equivalent, when measured against a reliable comparator (i.e., 2-minute scan segmentations). Equivalence bounds were determined based on the interquartile range of previous interobserver data from McDonald et al. (49) (**Appendix D, Table 1D**). The Python package statsmodels (53) was used to conduct the TOST analysis. Finally, metrics (DSC and ASD) were also calculated between OAR segmentations on ground-truth 6-minute images vs. synthetic 6-minute images and qualitatively compared to the previously established IOV values.

### Manual segmentation evaluation

In order to further investigate the acceptability of our synthetic images for use in segmentation workflows, we compared physician-generated manual OAR segmentations using ground-truth and synthetic 6-minute images. A subset of five HNC patients with a primary diagnosis of oropharyngeal cancer were used for the analysis. Three radiation oncologist observers provided segmentations for the analysis (D.E., Y.K., M.A.). Pairwise metrics (DSC and ASD) were calculated between all observers to determine the IOV for each image type (ground-truth, synthetic). Before comparison, OAR masks were resampled to the same image space as the ground-truth 6-minute image using a nearest neighbor interpolator. Paired TOST analysis was performed to determine if IOV of ground-truth and synthetic scans was equivalent; p-values less than 0.05 were considered statistically significant. Equivalence bounds for each OAR were based on the interquartile range of corresponding ground-truth images. Finally, for each observer, metrics (DSC and ASD) were calculated between OAR segmentations on ground-truth images vs. synthetic images and qualitatively compared to the corresponding ground-truth IOV.

### Visual Turing test

To determine whether synthetic images were visually distinguishable from ground-truth images by human expert observers, we implemented a visual Turing test inspired by Gooding et al. (54). A subset of five HNC patients with a primary diagnosis of oropharyngeal cancer with visible primary and nodal tumors on imaging were selected for the Turing test (the same patients used for the manual segmentation evaluation). Paired image representations were randomly generated for each slice, i.e., random allocation of images to either the left or right image. Four axial slices for six ROIs (parotid glands, submandibular glands, mandible, tumor, node) were selected for each case, leading to 100 paired images available for evaluation. Images were randomly shuffled before evaluation. Moreover, to ensure an equal comparison that is unbiased by arbitrary MRI voxel units, we applied a z-score normalization to each presented slice. Three radiation oncologists (D.E., Y.K., M.A.) were asked to provide their best guess of which image (left or right) was real (ground-truth) and synthetic (DL-generated). In addition, clinicians were asked to denote which image they preferred overall. Finally, clinicians were asked to provide comments on why they made their decision. The test was conducted twice: first with the raw DL outputs and 2 weeks later after applying a 3x3 sharpening kernel = [[0, -0.5, 0], [-0.5, 3,-0.5], [0, -0.5, 0]] to DL outputs. To statistically evaluate the Turing test results, for each observer we implemented a two one-sided test for two proportions using an expected proportion of 0.5 with equivalence bounds of -0.3 and 0.3; the function was derived from the TOSTER R package (55) and implemented in Python through the Rpy2 Python package (56). For all statistical analyses, p-values less than 0.05 were considered statistically significant.

### Qualitative evaluation of failure cases

We visually evaluated images of a select subset of cases by comparing ground-truth 6-minute scans and post-sharpened synthetic 6-minute scans. Low-, medium-, and high-performance example cases were selected on the basis of whether they were lower than, equal to, or higher than the median SSIM value of the external mask across all cases. Pixel-wise difference maps and SSIM maps between the normalized ground-truth and synthetic scans were generated to visualize uncertainties in relation to the DL synthesis process. For comparison, we also displayed the input 2-minute T2w scan for each case.

## Results

### Image similarity evaluation

**Table 2** shows the values of the various intensity metrics for the whole image and ROI evaluations. Two cases had surgical resections which obfuscated normal submandibular gland anatomy and prohibited the auto-segmentation algorithm from generating a large enough ROI for similarity analysis; therefore, these respective auto-segmented submandibular glands were not used. Generally, the whole image boasted the best median (interquartile range [IQR]) similarity metric values of 0.19 (0.05), 0.93 (0.03), and 33.14 (2.30) for MSE, SSIM, and PSNR, respectively. MSE, SSIM, and PSNR values slightly worsened when evaluated on the external mask to 0.41 (0.12), 0.80 (0.06), and 30.06 (1.32), respectively. Within the OARs, the best MSE and PSNR were for the mandible (1.16 [0.50] and 23.33 [1.83], respectively), the worst SSIM was 0.43 (0.12) for the left submandibular gland, and the worst PSNR was 18.02 (3.34) for the right submandibular gland. The brainstem simultaneously achieved the best SSIM of 0.66 (0.09) but the worst MSE of 4.99 (2.98) among the OARs. **Appendix E** shows the impact of applying various processing steps on similarity values and preliminary analyses on radiomic features.

**Table 2:** Image similarity metric results across the whole image and various subregions.

ROI	MSE	SSIM	PSNR
Whole	0.19 (0.05)	0.93 (0.03)	33.14 (2.30)
External	0.41 (0.12)	0.80 (0.06)	30.06 (1.32)
Mandible	1.16 (0.50)	0.56 (0.09)	23.33 (1.83)
Brainstem	4.99 (2.98)	0.66 (0.09)	19.17 (2.03)
Left Submandibular Gland	1.42 (0.79)	0.43 (0.12)	19.52 (3.56)
Right Submandibular Gland	1.44 (0.84)	0.48 (0.14)	18.02 (3.34)
Left Parotid Gland	1.40 (0.52)	0.46 (0.11)	18.87 (2.64)
Right Parotid Gland	1.20 (0.52)	0.52 (0.11)	19.38 (1.95)
Spinal Cord	1.98 (2.04)	0.51 (0.09)	22.56 (2.45)

Values presented are rounded up to two decimal places. MSE, mean squared error; SSIM, structural similarity index; PSNR, peak signal-to-noise ratio.

### Auto-segmentation evaluation

**Figure 3:**
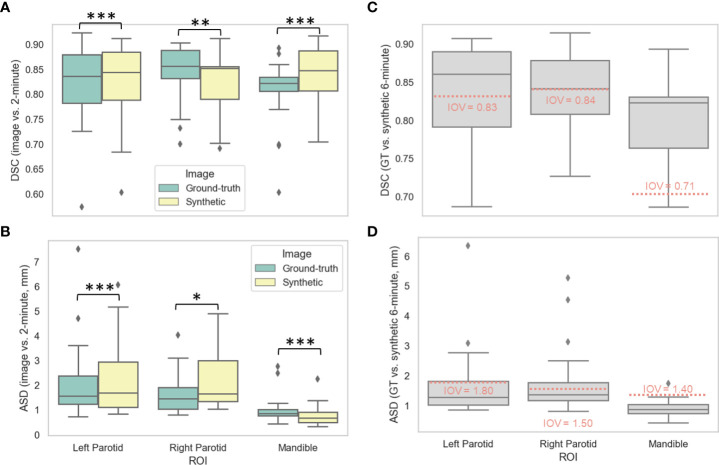
Auto-segmentation results. Auto-segmented organs at risk (left parotid gland, right parotid gland, and mandible) were generated on ground-truth ([GT], green) or synthetic (yellow) 6-minute images and compared against 2-minute images using the **(A)** Dice similarity coefficient (DSC) and **(B)** average surface distance (ASD). Segmentations generated on GT and synthetic images were then directly compared using the **(C)** DSC and **(D)** ASD. Stars above plot indicate paired two one-sided t-tests (equivalence test) level of significance: ns: p > 0.05; *: 0.005 < p <= 0.05; **: 0.0005 < p <= 0.005; ***: p<= 0.0005. Red dotted lines correspond to median interobserver variability (IOV) metric values derived from clinical experts from previous literature investigating 2-minute images.

**Figures 3A**, **B** show the distributions of the various auto-segmented OARs for the ground-truth images compared to the synthetic images for DSC and ASD, respectively. Only the right parotid gland, left parotid gland, and mandible were included in the analysis since they crossed previously recorded IOV cutoffs in ground-truth images (see details in **Appendix D**). One case had a right parotidectomy; therefore, it was excluded from the right parotid analysis. The median (IQR) DSC values and equivalence test p-values comparing ground-truth vs. synthetic OARs were 0.86 (0.06) vs. 0.85 (0.07) (p=0.001), 0.84 (0.10) vs. 0.84 (0.10) (p=1.08e-7), and 0.82 (0.03) vs. 0.85 (0.08) (p=3.48e-5) for the right parotid gland, left parotid gland, and mandible, respectively. The median (IQR) ASD values and equivalence test p-values comparing ground-truth vs. synthetic OARs were 1.45 (0.88) vs. 1.65 (1.65) (p=0.048), 1.56 (1.14) vs. 1.68 (1.83) (p=4.99e-5), and 0.86 (0.25) vs. 0.67 (0.41) (p=1.57e-7) for the right parotid gland, left parotid gland, and mandible, respectively. **Figures 3C**, **D** show the distributions of ground-truth vs. synthetic auto-segmented OAR overlap using DSC and ASD, respectively. Median metric values surpassed 2-minute IOV cutoffs for all investigated OARs. The median (IQR) DSC values were 0.84 (0.07), 0.86 (0.09), and 0.82 (0.07) for the right parotid gland, left parotid gland, and mandible, respectively. The median (IQR) ASD values were 1.36 (0.61), 1.27 (0.80), 0.87 (0.30) for the right parotid gland, left parotid gland, and mandible, respectively. Direct metric comparisons for the OARs that did not cross IOV cutoffs are shown in **Appendix D**.

### Manual segmentation evaluation

**Figure 4:**
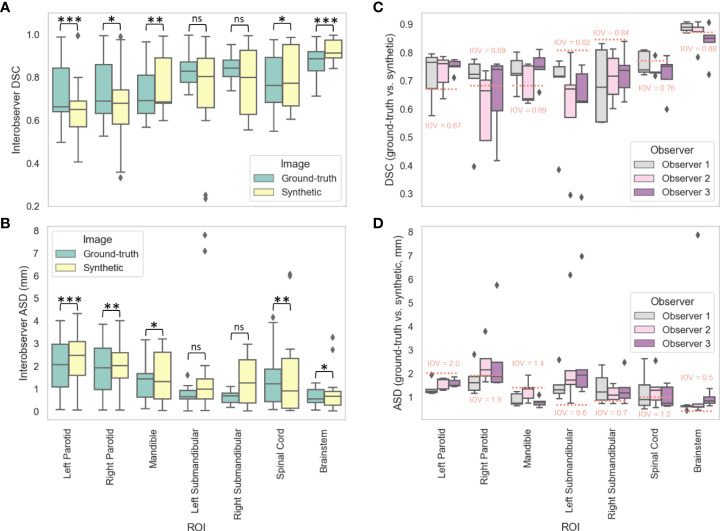
Manual segmentation results. For three clinician observers, manually segmented organs at risk (left parotid gland, right parotid gland, mandible, left submandibular gland, right submandibular gland, spinal cord, brainstem) were generated on ground-truth or synthetic 6-minute images; interobserver variability (IOV) values were calculated pairwise between each observer for ground-truth (green) and synthetic (yellow) images using the **(A)** Dice similarity coefficient (DSC) and **(B)** average surface distance (ASD). Segmentations generated on ground-truth and synthetic images were then compared for each observer using the **(C)** DSC and **(D)** ASD. Stars above plot indicate paired two one-sided t-tests (equivalence test) level of significance: ns: p > 0.05; *: 0.005 < p <= 0.05; **: 0.0005 < p <= 0.005; ***: p<= 0.0005. Red dotted lines correspond to median IOV metric values derived from clinical experts on ground-truth 6-minute images in this study.

**Figures 4A**, **B** show the distributions of the IOV values for the various manually segmented OARs for the ground-truth images compared to the synthetic images for DSC and ASD, respectively. The median (IQR) DSC IOV values and equivalence test p-values comparing ground-truth vs. synthetic OARs were 0.69 (0.23) vs. 0.65 (0.16) (p=0.007), 0.66 (0.20) vs. 0.65 (0.12) (p=6.44e-5), 0.69 (0.18) vs. 0.69 (0.21) (p=0.001), 0.84 (0.07) vs. 0.80 (0.30) (p=0.43), 0.83 (0.09) vs. 0.80 (0.23) (p=0.49), 0.89 (0.09) vs. 0.91 (0.08) (p=0.02), and 0.76 (0.21) vs. 0.77 (0.29) (p=2.85e-9) for the right parotid gland, left parotid gland, mandible, right submandibular gland, left submandibular gland, brainstem, and spinal cord, respectively. The median (IQR) ASD IOV values and equivalence test p-values comparing ground-truth vs. synthetic OARs were 1.93 (1.82) vs. 2.03 (1.12) (p=0.004), 2.07 (1.88) vs. 2.48 (1.50) (p=3.68e-6), 1.43 (1.04) vs. 1.31 (2.07) (p=0.007), 0.68 (0.41) vs. 1.27 (1.91) (p=0.88), 0.64 (0.38) vs. 0.99 (0.89) (p=0.84), 0.55 (0.57) vs. 0.67 (0.61) (p=0.04), and 1.23 (1.43) vs. 0.91 (2.21) (p=0.001) for the right parotid gland, left parotid gland, mandible, right submandibular gland, left submandibular gland, brainstem, and spinal cord, respectively. **Figures 4C**, **D** show the distributions of ground-truth vs. synthetic OAR overlap for each observer using DSC and ASD, respectively. Generally, median metric values surpassed IOV cutoffs for most observers for most OARs. Notable exceptions where IOV metric values were not crossed included the right parotid gland (DSC and ASD for observer 2), left submandibular gland (DSC and ASD for all observers), right submandibular gland (DSC and ASD for all observers), spinal cord (DSC for all observers, ASD for observer 2), and brainstem (DSC for observer 3, ASD for all observers).

### Visual Turing test

**Figure 5:**
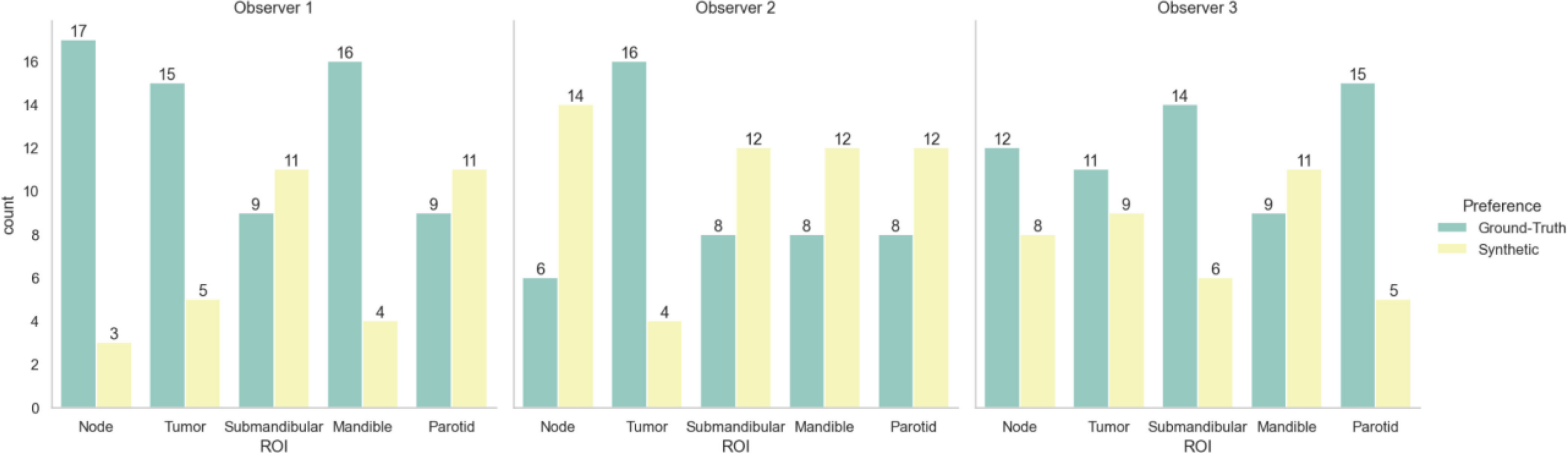
Clinician image preferences stratified by region represented in the presented image slice. Green bars correspond to ground-truth 6-minute MRI slices, while yellow bars correspond to synthetic 6-minute MRI slices.

**Table 3** shows the Turing test and clinician preference results. Significance testing for both proportions of images correctly identified and image preferences revealed equivalence between the ground-truth images and the synthetic images for all observers (p < 0.05). **Figure 5** stratifies the clinician preference results by the predominant ROI contained in each slice. All clinicians preferred the ground-truth for slices with primary tumors present, while there was no clear consensus for other regions. Observer comments for the Turing test generally focused on the ability to discriminate margins between structures (raw comments are shown in **Appendix F**). Additionally, in **Appendix F**, we show that without the application of the sharpening filter, clinicians made a clearer distinction between the synthetic and ground-truth images.

**Table 3:** Visual Turing test and image preference results for three physician expert observers.

Observer	% Correct	p-val	% Ground-Truth Preference	p-val
1	51	2.05e-05	66	0.02
2	56	3.29e-4	46	1.12e-4
3	59	1.37e-3	61	3.26e-3

Each observer was asked to determine the image identity of blinded paired ground-truth or synthetic 6-minute scan slices in a randomized fashion and provide their preference. Two one-sided tests for two proportions were applied to determine whether observer estimates were equivalent to chance.

### Qualitative evaluation

**Figure 6:**
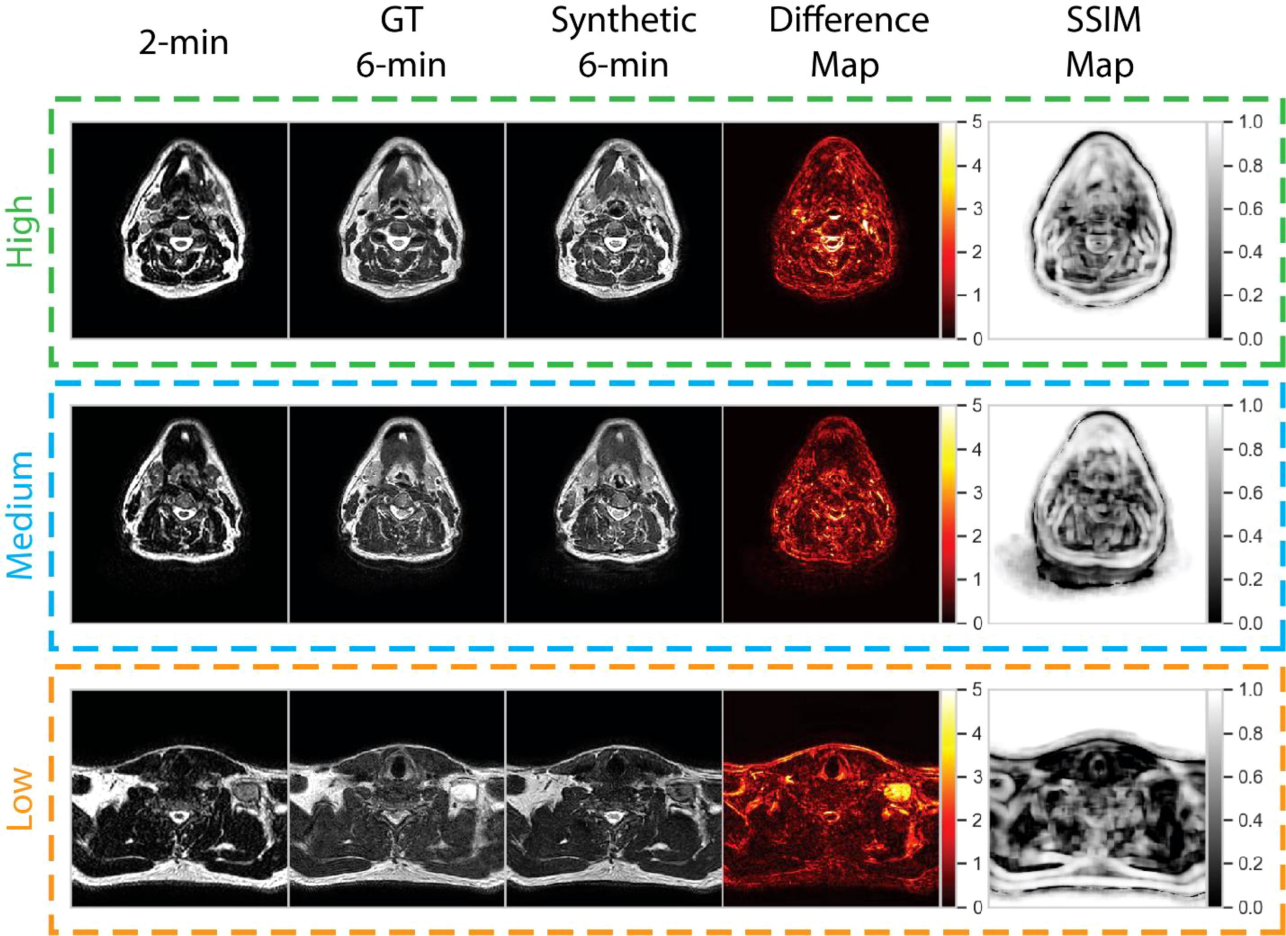
Qualitative evaluation of select cases from the test set. Cases correspond to high (green), medium (blue), and low (orange) performance relative to the median structural similarity index (SSIM) for the entire test set. Paired 2-minute scans, ground-truth 6-minute scans, synthetic 6-minute scans, absolute difference intensity maps between the ground-truth and synthetic 6-minute scans, and SSIM maps between the ground-truth and synthetic 6-minute scans are shown for each case. The high-, medium-, and low-performance cases correspond to patients with glandular (post-resection), oropharyngeal, and unknown (metastatic lymph node) primary tumors, respectively.

To help visualize image similarity results, we inspected paired ground-truth and synthetic images for a few select cases based on SSIM scores of the external mask in **Figure 6**. In general, larger differences in areas of relative hyperintensity present on both 2-minute scans and ground-truth 6-minute scans were found between the ground-truth and synthetic images, e.g., fat and cerebrospinal fluid. For the low-performance case (SSIM = 0.68), which corresponds to a patient with an unknown primary tumor, a large level VI lymph node demonstrated hyperintensity on the ground-truth 6-minute scan. However, the difference map demonstrated a large discrepancy in the nodal area on the synthetic scan, likely due to a lack of visible contrast on the 2-minute scan input. An inability to synthesize the borders of the vocal folds was also noted. Relative differences were minor for most other areas of the image. For the medium-performance (SSIM = 0.81) and high-performance (SSIM = 0.86) cases, which corresponded to patients with glandular and oropharyngeal primary tumors, respectively, there were no major deviations in specific regions with the exception of previously mentioned hyperintense areas. SSIM maps were generally correlated with difference maps for all cases and had lower similarity values for internal tissue structures and near tissue-background boundaries.

## Discussion

In this proof of principle study, we demonstrated the feasibility of using DL to reduce overall HNC MRI scan time by generating a 6-minute quality synthetic scan from a 2-minute scan input. A variety of evaluation techniques were performed, including similarity metric analysis, OAR segmentation, and a clinician-based visual Turing test, which demonstrate the reasonable quality of our synthetically generated scans and potential for integration into clinical workflows. To our knowledge, this is the first study investigating the impact of DL-based synthetic image generation in the context of decreased MRI scan time in HNC and the first study to investigate synthetic image acceptability for MRI-guided RT applications.

While it is difficult to directly compare metric values between different studies due to variability in datasets and model training, our models boast similar performance to current state-of-the-art methods in related literature. Notably, we ensured image normalization before metric calculation, but since MSE and PSNR are highly dependent on the scale of the intensity values investigated we avoid direct comparisons to other work. However, SSIM provides an intensity scale-invariant method of calculating similarity, which allows for a surface-level comparison with other investigations. To our knowledge, the only investigation of DL-generated synthetic MRI from input MRI sequences in HNC was performed by Li et al. (31). In their study, the authors sought to generate synthetic post-contrast T1-weighted (T1w) scans from pre-contrast T1w and T2w scans, which yielded average whole-image SSIM values of 0.88 in their test set for their best-performing model. Moreover, several studies synthesizing T1w or T2w scans also reported similar SSIM values, often in the range of 0.85-0.95 (22, 25, 29, 30). Therefore, it is encouraging that our whole-image SSIM values of 0.93 are comparable or even superior to those currently reported. It should be noted that metric values tended to worsen when evaluating specific subregions compared to the whole image. As seen in the SSIM maps of our qualitative analysis, SSIM tended to be lower for internal tissue regions. These regions may have greater intricate details that are difficult to replicate accurately. However, this trend was also noted in Li et al., where SSIM decreased from 0.86 in the whole image to 0.64 in the tumor region. Importantly, SSIM is also a function of the window size parameter, which is not currently standardized across synthetic image analysis studies. Interestingly, within the OAR regions investigated, the brainstem achieved the best SSIM but the worst MSE, indicating that the synthetic brainstem was structurally similar to the ground-truth brainstem but had a greater relative difference in intensity values than other OARs.

One of the main end-uses of anatomical MRIs in an ART workflow is for ROI segmentation (3). In proposed future workflows for MRI-guided ART, it is envisioned that anatomical sequences will be used to segment OARs, while functional sequences, such as diffusion-weighted and dynamic contrast-enhanced imaging, will provide greater useful information for segmenting target structures. Therefore, we have chosen to focus this study on investigating OAR-specific ROIs. Moreover, we have established physician preference for visualizing OARs on 6-minute scans compared to 2-minute scans in our supplementary analysis; therefore, it can be reasonably assumed that synthetic scans which demonstrate equivalent scan quality to a 6-minute scan would be clinically useful for OAR segmentation. While manual segmentation of OARs is still the current clinical standard, increasing effort has shown promising results for automatic OAR segmentation through DL methods (57). Our results indicate that certain auto-segmented OARs (parotid glands and mandible) are not significantly different in segmentation quality between ground-truth and synthetic scans. In essence, the previously trained auto-segmentation algorithm faced its own “artificial Turing Test” when using synthetic or ground-truth 6-minute scans as input. Moreover, direct comparison of auto-segmented OARs crossed previous IOV thresholds. Subsequently, it stands to reason that a synthetic 6-minute MRI may be able to replace a real 6-minute MRI for auto-segmenting these OARs. In addition to auto-segmentation based quality analysis, we have also included an investigation of manual segmentation for a subset of cases. IOV measured across 3 physician observers was found to be equivalent for all OARs with the exception of submandibular glands, which exhibited higher variability on synthetic images when compared to ground-truth images. When directly comparing segmentations performed on ground-truth with synthetic images, IOV thresholds were crossed for most observers across most OARs, but there were several notable exceptions, chief of which was particularly poor performance for submandibular gland segmentation. Therefore, while overall segmentation results are encouraging, there should still be caution in implementing synthetic images for OAR segmentation purposes.

Unlike CT imaging, where voxel-level quantitative information can be used in the RT workflow, e.g., dose calculations, MRIs are currently mainly utilized for human-level decision making, e.g., segmentation. Arguably, the most important facet of MRI quality in current RT workflows is clinician interpretation of image quality. Therefore, the passing of a clinician-based visual Turing test for synthetic 6-minute scans is paramount to determining the preliminary clinical utility of this technology. In aggregate across all observers, there was no major preference given to either the ground-truth or synthetic 6-minute images, indicating a similarity in the human interpretation of image quality. Moreover, observers were at most only able to correctly determine the true identity of the blinded image 59% of the time, compared to the assumed 50% if clinicians were randomly guessing. It should be noted that clinicians did have a slight preference for visualizing slices containing target volumes on ground-truth images. However, as previously noted, OAR regions are of greater interest in these anatomical sequences; therefore, whether this preference would be clinically meaningful could be debated.

Our qualitative analysis revealed that across most patients observed, there was a larger disparity in regions of relatively high intensity on ground-truth imaging, e.g., fat and cerebrospinal fluid. The larger absolute range of values may make it more difficult for the DL network to synthesize these regions precisely. Moreover, SSIM maps indicated model difficulties with approximating certain tissue and background boundaries, secondary to visually imperceptible signal differences, i.e., noise, in these areas. However, as the goal of this work is to improve clinician workflows, these perceived differences are likely not clinically significant. Moreover, these issues have been echoed in similar work (31). Consistent with clinician preferences, our DL model generally had difficulties in successfully synthesizing pathologic tissue, i.e., primary and nodal tumor volumes. This is likely secondary to the relative lack of representation of pathologic tissue in the training set since models are trained at a slice-by-slice level. Our models were trained using patients from a variety of HNC subsites (i.e., oropharynx, nasopharynx, glandular, etc.), so there was substantial heterogeneity in nodal appearance and location, which likely made model training difficult for these subregions. However, while the contrast in these nodal regions was often visibly different in synthetic images, their relative size, shape, and texture may remain similar to the ground-truth image. It has been suggested that geometrical properties, i.e., size, shape, and texture, are often particularly important for image segmentation (58, 59). Therefore, cases where tumor volume synthesis “fails” may not necessarily render these images clinically unusable, but additional research should be performed to verify these claims.

Previous work by McDonald et al. investigating the Unity online adaptive workflow in HNC patients has demonstrated total treatment times ranging from 31 to 85 minutes (5). This raises the important question of whether the ~4 minutes saved using our method is justified. While on a patient-by patient basis these time savings may not seem significant, it is important to note that patients are fixed in immobilization devices, often with intraoral mouth pieces, which may be claustrophobic and highly uncomfortable for patients (60). Therefore, presumably, every minute saved adds to an improvement in patient comfort during their already harrowing treatment process. Furthermore, an important merit of decreased image acquisition time is a reduced probability of voluntary or involuntary patient movement (61), therefore our method could potentially save time by avoiding repeated imaging induced by motion artifact. Moreover, given current treatment times, it is common to treat 6-8 patients per day at our institution, so over the course of one day, these time savings could accumulate and lead to another full treatment that could be delivered during clinic hours. In addition, it warrants noting that image acquisition is the first step in treatment. Accordingly, no other imaging or planning activities can take place while the initial MRI sequence is acquired, unlike other steps in the workflow. Therefore, efficiency is further improved, and additional compounding timesaving’s may be possible due to the shorter initial acquisition times. Finally, while our method has only been applied to the standard 6-minute sequence utilized in the Unity workflow, future studies using analogous methods could be applied to superior high-quality MRI sequences acquired over periods infeasible for the current workflow (e.g. > 6-minutes). In a similar vein, future work could also investigate the use of MRI sequences acquired over 1-minute or less to generate synthetic high-quality sequences, thereby further improving time savings.

Our study is not without limitations. Although our total number of unique image sets was larger than most in the existing HNC synthetic imaging literature (16, 18, 20, 21, 24, 31), our model training and evaluation was limited to a small cohort from the same institution. However, since model training occurred on a slice-by-slice basis, we utilized on the order of ~20,000 training data points, which allowed us to leverage DL approaches effectively. Moreover, we only tested one DL approach; several architectural modifications have been proposed that could improve our models in terms of similarity metric performance (31). These architectural improvements may be particularly salient for improving synthesis of specific regions such as challenging OARs that were unable to be appropriately manually segmented by clinicians, e.g., submandibular glands. A further limitation of our study is that we used a previously trained DL OAR auto-segmentation model that was developed for 2-minute T2w scans, which limited its generalizability to 6-minute T2w scans. However, the performance of the model on the 6-minute scans was above the expected clinical interobserver variability for several OARs (both when considering measurements previously obtained on 2-minute scans and new measurements performed on 6-minute scans) and we were therefore confident in its use for the analysis; interestingly, these results offer evidence for the generalizability of MRI auto-segmentation models, but future studies should further investigate how to optimally translate models developed for different sequences. On a related note, while we have not utilized auto-segmented structures with obfuscated anatomy (e.g., surgically resected glands) in our analysis, additional studies should examine model performance on outlier cases with irregular anatomical presentations. Additionally, it warrants mentioning that the raw outputs of the DL model were often blurrier than their ground-truth counterparts. This effect has been widely documented in synthetic image studies (15, 20, 22, 24, 25, 30), often driven by a lower spatial resolution input image up-sampled to match a higher spatial resolution output image. While this slight blurring effect is unlikely to affect underlying image quality (as evidenced by metric performance), in supplementary analyses we demonstrated that this difference was perceived by the clinicians and added bias to the analysis. Therefore, we applied a sharpening filter to synthetic images for the Turing test to remove bias associated with blurry DL outputs, which yielded improved results. While the application of a sharpening filter serves as an effective mitigation technique, as shown in the supplementary analysis, it slightly decreased similarity metric performance. Therefore more sophisticated approaches to decrease image blurriness without the cost of image quality should be investigated, such as the use of super-resolution DL networks (62). Furthermore, the role of imaging biomarkers, i.e., radiomics (63), is predicted to play an increasing role in MRI-guided ART (3, 4). While we have provided minor supplementary analyses on this issue, future work should investigate the feasibility of using synthetic images for radiomic-related analyses. Finally, dosimetric differences between ground-truth and synthetic images have not been investigated in this work, but should be the focus of future, ideally prospective, studies.

## Conclusions

In summary, using 2-minute MRI inputs, we designed a CycleGAN DL model to generate synthetic scans that were similar to ground-truth 6-minute scans. As evidenced in quantitative and qualitative analysis, our synthetic scans are of comparable quality to ground-truth 6-minute scans, but particular caution should be noted for segmentation applications of certain OARs such as submandibular glands. This model could act as a starting point for the generation of high-quality scans at a reduced acquisition time, thereby improving patient comfort and scanner availability in an MRI-guided ART workflow. Future studies should include external validation of our model, DL architectural improvements, and investigating synthetic images in the context of imaging biomarkers. Moreover, while we study the generation of high-quality output images from standard 2-minute input images, potentially these methods could be extrapolated to input images acquired over an even shorter period (e.g., < 1-minute acquisition) and with higher quality output images (e.g., > 6-minute acquisition) to further improve time-savings.

## Data availability statement

The datasets presented in this study can be found in online repositories. The names of the repository/repositories and accession number(s) can be found below: 10.6084/m9.figshare.20099252.

## Ethics statement

The studies involving human participants were reviewed and approved by the University of Texas MD Anderson Cancer Center’s Institutional Review Board. The patients/participants provided their written informed consent to participate in this study.

## Author contributions

Study Conceptualization: JX, NO’C, JC, BM, CF, MN, AM; Data collection: DE, YK, MA, BM, SA, CS, KP; Experiments: KW, MN, JX, NO’C, DT, NC; Analysis: KW, JX, NO’C, TS, RH, MN; Manuscript Writing: KW, JX; Manuscript Editing: KW, DE, YK, MA, TS, AM.

## Funding

This work was supported by the National Institutes of Health (NIH)/National Cancer Institute (NCI) through a Cancer Center Support Grant (P30CA016672-44). KW is supported by the Dr. John J. Kopchick Fellowship through The University of Texas MD Anderson UTHealth Graduate School of Biomedical Sciences, the American Legion Auxiliary Fellowship in Cancer Research, and an NIH/National Institute for Dental and Craniofacial Research (NIDCR) F31 fellowship (1 F31DE031502-01). TS is supported by The University of Texas Health Science Center at Houston Center for Clinical and Translational Sciences TL1 Program (TL1 TR003169). MN is supported by an NIH grant (R01DE028290-01). CF received funding from the NIH/NIDCR (1R01DE025248-01/R56DE025248); an NIH/NIDCR Academic-Industrial Partnership Award (R01DE028290); the National Science Foundation (NSF), Division of Mathematical Sciences, Joint NIH/NSF Initiative on Quantitative Approaches to Biomedical Big Data (QuBBD) Grant (NSF 1557679); the NIH Big Data to Knowledge (BD2K) Program of the NCI Early Stage Development of Technologies in Biomedical Computing, Informatics, and Big Data Science Award (1R01CA214825); the NCI Early Phase Clinical Trials in Imaging and Image-Guided Interventions Program (1R01CA218148); an NIH/NCI Pilot Research Program Award from the UT MD Anderson CCSG Radiation Oncology and Cancer Imaging Program (P30CA016672); an NIH/NCI Head and Neck Specialized Programs of Research Excellence (SPORE) Developmental Research Program Award (P50CA097007); and the National Institute of Biomedical Imaging and Bioengineering (NIBIB) Research Education Program (R25EB025787).

## Acknowledgments

We thank Ms. Ann Sutton from the Editing Services Group at The University of Texas MD Anderson Cancer Center Research Medical Library for editing this article. The authors also acknowledge the following individuals for their contributions to the NIH-funded academic-industrial partnership grant (R01DE028290) that funded this work and for their general support and feedback regarding this project: Spencer Marshall, Hafid Akhiat, Michel Moreau, Edyta Bubula-Rehm, Chunhua Men, and Etienne Lessard of Elekta and Alex Dresner of Philips.

## Conflict of interest

CF has received direct industry grant support, speaking honoraria, and travel funding from Elekta AB. JX, NO’C, DT, NC, and JC are employees of Elekta AB.

The remaining authors declare that the research was conducted in the absence of any commercial or financial relationships that could be construed as a potential conflict of interest.

## Publisher’s note

All claims expressed in this article are solely those of the authors and do not necessarily represent those of their affiliated organizations, or those of the publisher, the editors and the reviewers. Any product that may be evaluated in this article, or claim that may be made by its manufacturer, is not guaranteed or endorsed by the publisher.

## References

[1] Rettig EM D’Souza. Epidemiology of head and neck cancer. Surg Oncol Clin N Am (2015) 24:379–96. doi: 10.1016/j.soc.2015.03.001 25979389

[2] Alterio D Marvaso G Ferrari A Volpe S Orecchia R Jereczek-Fossa BA Modern radiotherapy for head and neck cancer. Semin Oncol (2019) 46:233–45. doi: 10.1053/j.seminoncol.2019.07.002 31378376

[3] Mulder SL Heukelom J McDonald BA Van Dijk L Wahid KA Sanders K. MR-guided adaptive radiotherapy for OAR sparing in head and neck cancers. Cancers (2022) 14:1909. doi: 10.3390/cancers14081909 35454816 PMC9028510

[4] Kiser KJ Smith BD Wang J Fuller CD. “Après mois, le déluge”: Preparing for the coming data flood in the MRI-guided radiotherapy era. Front Oncol (2019) 9:983. doi: 10.3389/fonc.2019.00983 31632914 PMC6779062

[5] McDonald BA Vedam S Yang J Wang J Castillo P Lee B. Initial feasibility and clinical implementation of daily MR-guided adaptive head and neck cancer radiation therapy on a 1.5T MR-linac system: Prospective r-IDEAL 2a/2b systematic clinical evaluation of technical innovation. Int J Radiat Oncol Biol Phys (2021) 109:1606–18. doi: 10.1016/j.ijrobp.2020.12.015 PMC7965360 33340604

[6] Naser MA van Dijk LV He R Wahid KA Fuller CD. Tumor segmentation in patients with head and neck cancers using deep learning based-on multi-modality PET/CT images. Head Neck Tumor Segmentation (2021) 12603:85–98. doi: 10.1007/978-3-030-67194-5_10 33724743 PMC7929493

[7] Taku N Wahid KA van Dijk LV Sahlsten J Jaskari J Kaski K. Auto-detection and segmentation of involved lymph nodes in HPV-associated oropharyngeal cancer using a convolutional deep learning neural network. Clin Transl Radiat Oncol (2022) 36:47–55. doi: 10.1101/2022.01.19.22269566 PMC9240370 35782963

[8] Wahid KA Ahmed S He R van Dijk LV Teuwen J McDonald BA. Evaluation of deep learning-based multiparametric MRI oropharyngeal primary tumor auto-segmentation and investigation of input channel effects: Results from a prospective imaging registry. Clin Transl Radiat Oncol (2022) 32:6–14. doi: 10.1016/j.ctro.2021.10.003 34765748 PMC8570930

[9] Naser MA Wahid KA Grossberg AA Olson B Jain R El-Habashy D. Deep learning auto-segmentation of cervical neck skeletal muscle for sarcopenia analysis using pre-therapy CT in patients with head and neck cancer. Front Oncol (2022) 12. doi: 10.1101/2021.12.19.21268063 PMC9366009 35965493

[10] Naser MA Wahid KA van Dijk LV He R Abdelaal MA Dede C. Head and neck cancer primary tumor auto segmentation using model ensembling of deep learning in PET/CT images. Head Neck Tumor Segmentation Outcome Prediction. (2022) 13209:121–33. doi: 10.1007/978-3-030-98253-9_11 PMC8991449 35399869

[11] Naser MA Deen MJ. Brain tumor segmentation and grading of lower-grade glioma using deep learning in MRI images. Comput Biol Med (2020) 121:103758. doi: 10.1016/j.compbiomed.2020.103758 32568668

[12] Wahid KA He R Dede C Mohamed ASR Abdelaal MA van Dijk LV. Combining tumor segmentation masks with PET/CT images and clinical data in a deep learning framework for improved prognostic prediction in head and neck squamous cell carcinoma. Head Neck Tumor Segmentation Outcome Prediction. (2022) 13209:300–7. doi: 10.1007/978-3-030-98253-9_28 PMC8991448 35399870

[13] Naser MA Wahid KA Mohamed ASR Abdelaal MA He R Dede C. Progression free survival prediction for head and neck cancer using deep learning based on clinical and PET/CT imaging data. Head Neck Tumor Segmentation Outcome Prediction. (2022) 13209:287–99. doi: 10.1007/978-3-030-98253-9_27 PMC8991450 35399868

[14] Kim S-W Shin H-J Hwang J-H Shin J-S Park S-K Kim J-Y. Image similarity evaluation of the bulk-density-assigned synthetic CT derived from MRI of intracranial regions for radiation treatment. PLoS One (2017) 12:e0185082. doi: 10.1371/journal.pone.0185082 28926610 PMC5605009

[15] Jin C-B Kim H Liu M Jung W Joo S Park E. Deep CT to MR synthesis using paired and unpaired data. Sensors (2019) 19:2361. doi: 10.3390/s19102361 PMC6566351 31121961

[16] Wang Y Liu C Zhang X Deng W. Synthetic CT generation based on T2 weighted MRI of nasopharyngeal carcinoma (NPC) using a deep convolutional neural network (DCNN). Front Oncol (2019) 9:1333. doi: 10.3389/fonc.2019.01333 31850218 PMC6901977

[17] Lerner M Medin J Jamtheim Gustafsson C Alkner S Siversson C Olsson LE. Clinical validation of a commercially available deep learning software for synthetic CT generation for brain. Radiat Oncol (2021) 16:66. doi: 10.1186/s13014-021-01794-6 33827619 PMC8025544

[18] Olin AB Thomas C Hansen AE Rasmussen JH Krokos G Urbano TG. Robustness and generalizability of deep learning synthetic computed tomography for positron emission Tomography/Magnetic resonance imaging–based radiation therapy planning of patients with head and neck cancer. Adv Radiat Oncol (2021) 6:100762. doi: 10.1016/j.adro.2021.100762 34585026 PMC8452789

[19] Edmund JM Nyholm T. A review of substitute CT generation for MRI-only radiation therapy. Radiat Oncol (2017) 12:28. doi: 10.1186/s13014-016-0747-y 28126030 PMC5270229

[20] Olin AB Hansen AE Rasmussen JH Ladefoged CN Berthelsen AK Håkansson K. Feasibility of multiparametric positron emission Tomography/Magnetic resonance imaging as a one-stop shop for radiation therapy planning for patients with head and neck cancer. Int J Radiat OncologyBiologyPhysics (2020) 108:1329–38. doi: 10.1016/j.ijrobp.2020.07.024 32682955

[21] Olin AB Hansen AE Rasmussen JH Jakoby B Berthelsen AK Ladefoged CN. Deep learning for Dixon MRI-based attenuation correction in PET/MRI of head and neck cancer patients. EJNMMI Phys (2022) 9:20. doi: 10.1186/s40658-022-00449-z 35294629 PMC8927520

[22] Li W Li Y Qin W Liang X Xu J Xiong J. Magnetic resonance image (MRI) synthesis from brain computed tomography (CT) images based on deep learning methods for magnetic resonance (MR)-guided radiotherapy. Quantitative Imaging Med Surg (2020) 10:1223–36. doi: 10.21037/qims-19-885 PMC7276358 32550132

[23] Kieselmann JP Fuller CD Gurney-Champion OJ Oelfke U. Cross-modality deep learning: Contouring of MRI data from annotated CT data only. Med Phys (2021) 48:1673–84. doi: 10.1002/mp.14619 PMC8058228 33251619

[24] Lei Y Wang T Harms J Fu Y Dong X Curran WJ. CBCT-based synthetic MRI generation for CBCT-guided adaptive radiotherapy. In: Artificial intelligence in radiation therapy. Springer International Publishing (2019). p. 154–61.

[25] Hu N Zhang T Wu Y Tang B Li M Song B. Detecting brain lesions in suspected acute ischemic stroke with CT-based synthetic MRI using generative adversarial networks. Ann Transl Med (2022) 10:35. doi: 10.21037/atm-21-4056 35282087 PMC8848363

[26] Dai X Lei Y Wang T Zhou J Curran WJ Liu T. “Synthetic MRI-aided multi-organ segmentation in head-and-neck cone beam CT.” in: Medical Imaging 2021: Image-Guided Procedures, Robotic Interventions, and Modeling. (2021) 11598:438–43. SPIE, 2021. doi: 10.1117/12.2581128

[27] Diller G-P Vahle J Radke R Vidal MLB Fischer AJ Bauer UMM. Utility of deep learning networks for the generation of artificial cardiac magnetic resonance images in congenital heart disease. BMC Med Imaging (2020) 20:1–8. doi: 10.1186/s12880-020-00511-1 PMC7542728 33032536

[28] Han C Hayashi H Rundo L Araki R Shimoda W Muramatsu S. (2018). “GAN-based synthetic brain MR image generation.” In: 2018 IEEE 15th international symposium on biomedical imaging (ISBI 2018). pp. 734–738. IEEE.

[29] Moya-Sáez E Peña-Nogales Ó de Luis-GarcíaR Alberola-López C. A deep learning approach for synthetic MRI based on two routine sequences and training with synthetic data. Comput Methods Programs BioMed (2021) 210:106371. doi: 10.1016/j.cmpb.2021.106371 34525411

[30] Osman AFI Tamam NM. Deep learning-based convolutional neural network for intramodality brain MRI synthesis. J Appl Clin Med Phys (2022) 23:e13530. doi: 10.1002/acm2.13530 35044073 PMC8992958

[31] Li W Xiao H Li T Ren G Lam S Teng X. Virtual contrast-enhanced magnetic resonance images synthesis for patients with nasopharyngeal carcinoma using multimodality-guided synergistic neural network. Int J Radiat OncologyBiologyPhysics (2022) 112:1033–44. doi: 10.1016/j.ijrobp.2021.11.007 34774997

[32] Goodfellow I Pouget-Abadie J Mirza M Xu B Warde-Farley D Ozair S. Generative adversarial nets. Commun ACM (2020) 63 (11):139–44.

[33] Zhu J-Y Park T Isola P Efros AA. “Unpaired image-to-image translation using cycle-consistent adversarial networks.” In: Proceedings of the IEEE international conference on computer vision. (2017). pp. 2223–2232.

[34] Abadi M Agarwal A Barham P Brevdo E Chen Z Citro C. TensorFlow: Large-scale machine learning on heterogeneous distributed systems. arXiv (2016).

[35] Johnson J Alahi A Fei-Fei L. Perceptual losses for real-time style transfer and super-resolution. In: Computer vision – ECCV 2016. Springer International Publishing (2016). p. 694–711.

[36] Isola P Zhu J-Y Zhou T Efros AA. “Image-to-image translation with conditional adversarial networks.” In: Proceedings of the IEEE conference on computer vision and pattern recognition. (2017). pp. 1125–1134.

[37] Li C Wand M. Precomputed real-time texture synthesis with markovian generative adversarial networks. In: Computer vision – ECCV 2016. Springer International Publishing (2016). p. 702–16.

[38] Ledig C Theis L Huszár F Caballero J Cunningham A Acosta A. (2017). “Photo-realistic single image super-resolution using a generative adversarial network.” In: Proceedings of the IEEE conference on computer vision and pattern recognition. (2017). pp. 4681–90.

[39] Maes F Collignon A Vandermeulen D Marchal G Suetens P. Multimodality image registration by maximization of mutual information. IEEE Trans Med Imaging (1997) 16:187–98. doi: 10.1109/42.563664 9101328

[40] Reinhold JC Dewey BE Carass A Prince JL. Evaluating the impact of intensity normalization on MR image synthesis. Proc SPIE Int Soc Opt Eng (2019) 10949. doi: 10.1117/12.2513089 PMC6758567 31551645

[41] Tustison NJ Avants BB Cook PA Zheng Y Egan A Yushkevich PA. N4ITK: improved N3 bias correction. IEEE Trans Med Imaging (2010) 29:1310–20. doi: 10.1109/TMI.2010.2046908 PMC3071855 20378467

[42] Van Rossum G Drake FL. Python 3 reference manual. Scotts Valley CA: CreateSpace (2009).

[43] Anderson BM Wahid KA Brock KK. Simple Python module for conversions between DICOM images and radiation therapy structures, masks, and prediction arrays. Pract Radiat Oncol (2021) 11(3):226–9. doi: 10.1016/j.prro.2021.02.003 PMC8102371 33607331

[44] van der Walt S Colbert SC Varoquaux G. The NumPy array: A structure for efficient numerical computation. Computing Sci Eng (2011) 13:22–30. doi: 10.1109/MCSE.2011.37

[45] van der Walt S Schönberger JL Nunez-Iglesias J Boulogne F Warner JD Yager N. Scikit-image: image processing in Python. Peer J (2014) 2:e453. doi: 10.7287/peerj.preprints.336v2 25024921 PMC4081273

[46] Venkataramanan AK Wu C Bovik AC Katsavounidis I Shahid Z. A hitchhiker’s guide to structural similarity. IEEE Access (2021) 9:28872–96. doi: 10.1109/access.2021.3056504

[47] Otsu N. A threshold selection method from gray-level histograms. IEEE Trans Syst Man Cybern (1979) 9:62–6. doi: 10.1109/TSMC.1979.4310076

[48] Wahid KA He R McDonald BA Anderson BM Salzillo T Mulder S. Intensity standardization methods in magnetic resonance imaging of head and neck cancer. phiRO (2021) 2:88–93. 10.1016/j.phro.2021.11.001 PMC8607477 34849414

[49] McDonald BA Cardenas C O’Connell N Ahmed S Naser MA Wahid KA. Investigation of autosegmentation techniques on T2-weighted MRI for off-line dose reconstruction in MR-linac adapt to position workflow for head and neck cancers. medRxiv (2021). doi: 10.1101/2021.09.30.21264327 PMC10799175 37475466

[50] Sherer MV Lin D Elguindi S Duke S Tan L-T Cacicedo J. Metrics to evaluate the performance of auto-segmentation for radiation treatment planning: A critical review. Radiother Oncol (2021) 160:185–91. doi: 10.1016/j.radonc.2021.05.003 PMC9444281 33984348

[51] Nikolov S Blackwell S Zverovitch A Mendes R Livne M De Fauw J. Clinically applicable segmentation of head and neck anatomy for radiotherapy: Deep learning algorithm development and validation study. J Med Internet Res (2021) 23:e26151. doi: 10.2196/26151 34255661 PMC8314151

[52] Schuirmann DJ. A comparison of the two one-sided tests procedure and the power approach for assessing the equivalence of average bioavailability. J Pharmacokinet Biopharm (1987) 15:657–80. doi: 10.1007/BF01068419 3450848

[53] Skipper S Perktold J. “Statsmodels: Econometric and statistical modeling with python.” In: Proceedings of the 9th Python in Science Conference. (2010). vol. 57, no. 61, pp. 10–25080.

[54] Gooding MJ Smith AJ Tariq M Aljabar P Peressutti D van der Stoep J. Comparative evaluation of autocontouring in clinical practice: A practical method using the Turing test. Med Phys (2018) 45:5105–15. doi: 10.1002/mp.13200 30229951

[55] Lakens D Lakens MD. Package ‘TOSTER.’ (2018). Available at: http://mirror.salud.gob.sv/cran/web/packages/TOSTER/TOSTER.pdf.

[56] Gautier L. (2019). Available at: https://rpy2.bitbucket.io.

[57] Vrtovec T Močnik D Strojan P Pernuš F Ibragimov B. Auto-segmentation of organs at risk for head and neck radiotherapy planning: From atlas-based to deep learning methods. Med Phys (2020) 47:e929–50. doi: 10.1002/mp.14320 32510603

[58] Alzubaidi M Balasubramanian V Patel A Panchanathan S Black JA Jr. What catches a radiologist’s eye? a comprehensive comparison of feature types for saliency prediction. Med Imaging 2010: Computer-Aided Diagnosis. SPIE (2010) 7624:262–71. doi: 10.1117/12.844508

[59] Alexander RG Waite S Macknik SL Martinez-Conde S. What do radiologists look for? advances and limitations of perceptual learning in radiologic search. J Vis (2020) 20:17. doi: 10.1167/jov.20.10.17 PMC7571277 33057623

[60] Loja MAR Craveiro DS Vieira L Sousa E Rodrigues JA Portal RJF. Radiotherapy-customized head immobilization masks: from modeling and analysis to 3D printing. Nucl Sci Tech (2019) 30:1–16. doi: 10.1007/s41365-019-0667-2

[61] Zaitsev M Maclaren J Herbst M. Motion artifacts in MRI: A complex problem with many partial solutions. J Magn Reson Imaging (2015) 42:887–901. doi: 10.1002/jmri.24850 25630632 PMC4517972

[62] Li Y Sixou B Peyrin F. A review of the deep learning methods for medical images super resolution problems. IRBM (2021) 42:120–33. doi: 10.1016/j.irbm.2020.08.004

[63] Aerts HJWL Velazquez ER Leijenaar RTH Parmar C Grossmann P Carvalho S. Decoding tumour phenotype by noninvasive imaging using a quantitative radiomics approach. Nat Commun (2014) 5:4006. doi: 10.1038/ncomms5006 24892406 PMC4059926

