# Supplementary material for: Deep-learning-based generation of synthetic 6-minute MRI from 2-minute MRI for use in head and neck cancer radiotherapy
Source: Front Oncol. 2022 Nov 8;12:975902. doi: 10.3389/fonc.2022.975902 (PMC9679225; doi:10.3389/fonc.2022.975902)
Supplement: Supplementary file 1 [file DataSheet_1.pdf]

Appendices

Appendix A. Clinical Data

Table A1. Patient demographic characteristics. CUP = cancer of unknown primary.

Characteristic	Train (n=35)	Test (n=18)
Age (median, range)	63 (22-81)	62 (48-83)
Sex		
Male	28	17
Female	7	1
Race		
White	34	15
Other	1	3
Tumor site		
Oropharynx	18	8
Oral	3	2
Nasopharynx	3	1
Larynx	5	3
Node (CUP)	1	3
Volunteer	3	0
Gland	1	1
Other	1	0
T stage		
Tx, 0, or NA	5	5
1	8	4
2	12	6
3	3	0
4	7	3
N stage		

Nx, 0, or NA	16	4
1	9	6
2	9	5
3	1	3
M stage		
0 or NA	33	15
1	2	3

Appendix B. Additional Deep Learning Details

A DL generative adversarial neural network (GAN) (1) model based on the CycleGAN architecture (2) using paired T2w 2-minute and T2w 6-minute scans was implemented in Tensorflow (3). Specifically, our model implementation draws inspiration from work performed by Johnson et al. (4), which showed promising results for image translation. The overall structure of the generative networks is based on the classic 2D Resnet encoder-decoder structure, which consists of one convolution block with a 7x7 filter and stride size of 1, two convolution blocks with 3x3 filters and stride sizes of 2, 12 residual blocks with 3x3 filters and stride sizes of 1, two corresponding up-sampling convolution blocks with 3x3 filters and stride sizes of 1, and one convolutional block with a 7x7 filter and a stride size of 1; a tanh function was used for the final prediction output. The discriminator network is a PatchGAN (5–7) containing six convolutional layers: the first four layers were composed of 4x4 filters with stride sizes of 2, while the last two layers were composed of 4x4 filters with a stride size of 1. The discriminator aims to determine whether the 142x142 image patches obtained from either generators or true images are real or fake. Similar to the original CycleGAN study (2), leaky rectified linear unit activation functions and instance normalization were used throughout the generators and discriminators. The input and output image size for the generators was 512x512 after padding, and the output patch size of the discriminators was 32x32. **Figure B1** shows an overview of the DL architecture components. To obtain better quantitative synthetic results, we adopted an additional mutual information loss in addition to the original adversarial and cycle-consistency losses. The mutual information term (8) was calculated between the synthetic and the ground-truth images, and the negative mutual information was minimized. The weight parameters for the adversarial loss, cycle-consistency loss, and mutual information loss were set to 1, 10, and 1, respectively. The learning rate was fixed as 2e-4 for the first half of all training epochs and was linearly decayed for the second half of training; 200 total training epochs were used.

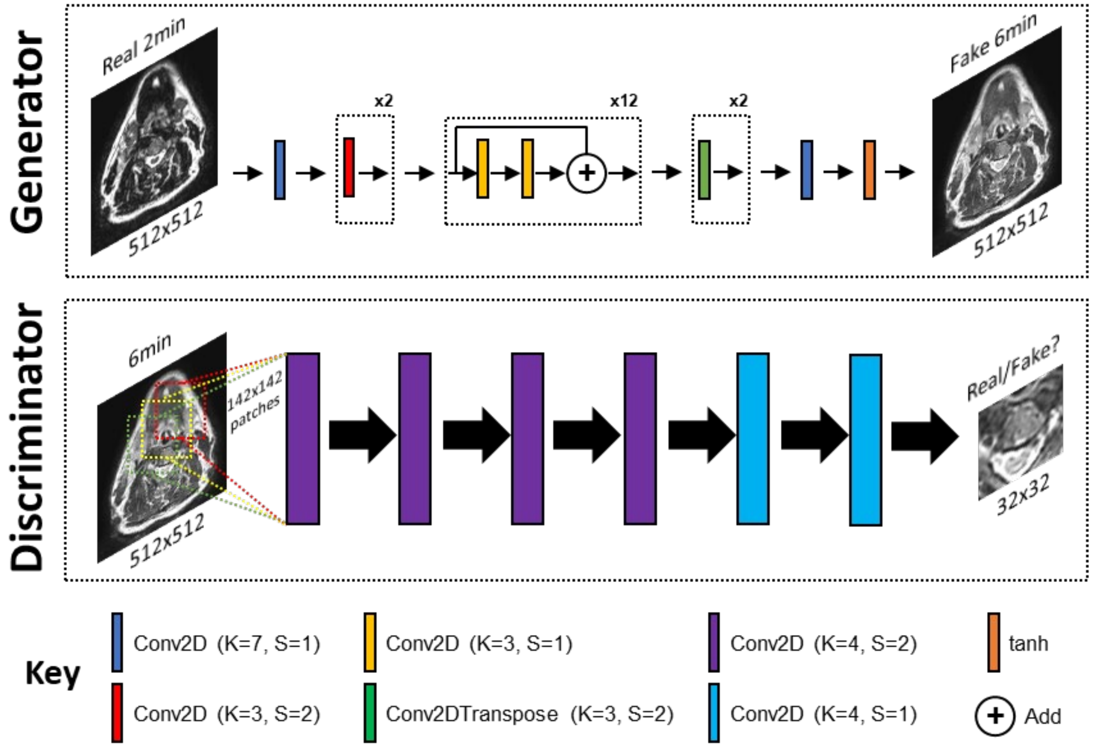

Figure B1. Overview of CycleGAN DL architecture. The general architecture consisted of a generator network (top) and PatchGAN discriminator network (bottom). Layers are represented as colored rectangles where K = kernel size and S = stride size. Each layer is followed by instance normalization and a leaky rectified linear unit activation function.

Appendix C. 6-minute MRI vs. 2-minute MRI Initial Survey

As initial motivation towards developing a synthetic MRI deep learning model, we created a survey to gauge physician preferences for ground-truth 2-minute vs. ground-truth 6-minute scans. Neuroimaging Informatics Technology Initiative (NIfTI) formatted ground-truth 2-minute scans and 6-minute scans of the 18 test cases were randomly relabeled as either “Image A” or “Image B”. These blinded images were provided to four physician observers to be visualized in 3D Slicer (9). Observers were free to alter the window width and level at their discretion. Observers were instructed to document their preference (“Image A” or “Image B”) in a spreadsheet for a set of regions of interest (ROI)s: right parotid gland, left parotid gland, left submandibular gland, right submandibular gland, spinal cord, brainstem, mandible, primary tumor, and metastatic lymph node(s). Not all images had all ROIs present (i.e., some patients had gland or tumor resections). Observer preferences were remapped to the original image identifiers to determine which observers preferred 6-minute scans vs. 2-minute scans for each ROI (**Figure C1**). With the exception of one observer, who preferred glandular structures on 2-minute scans, all observers overwhelmingly preferred 6-minute scans over 2-minute scans for all ROIs.

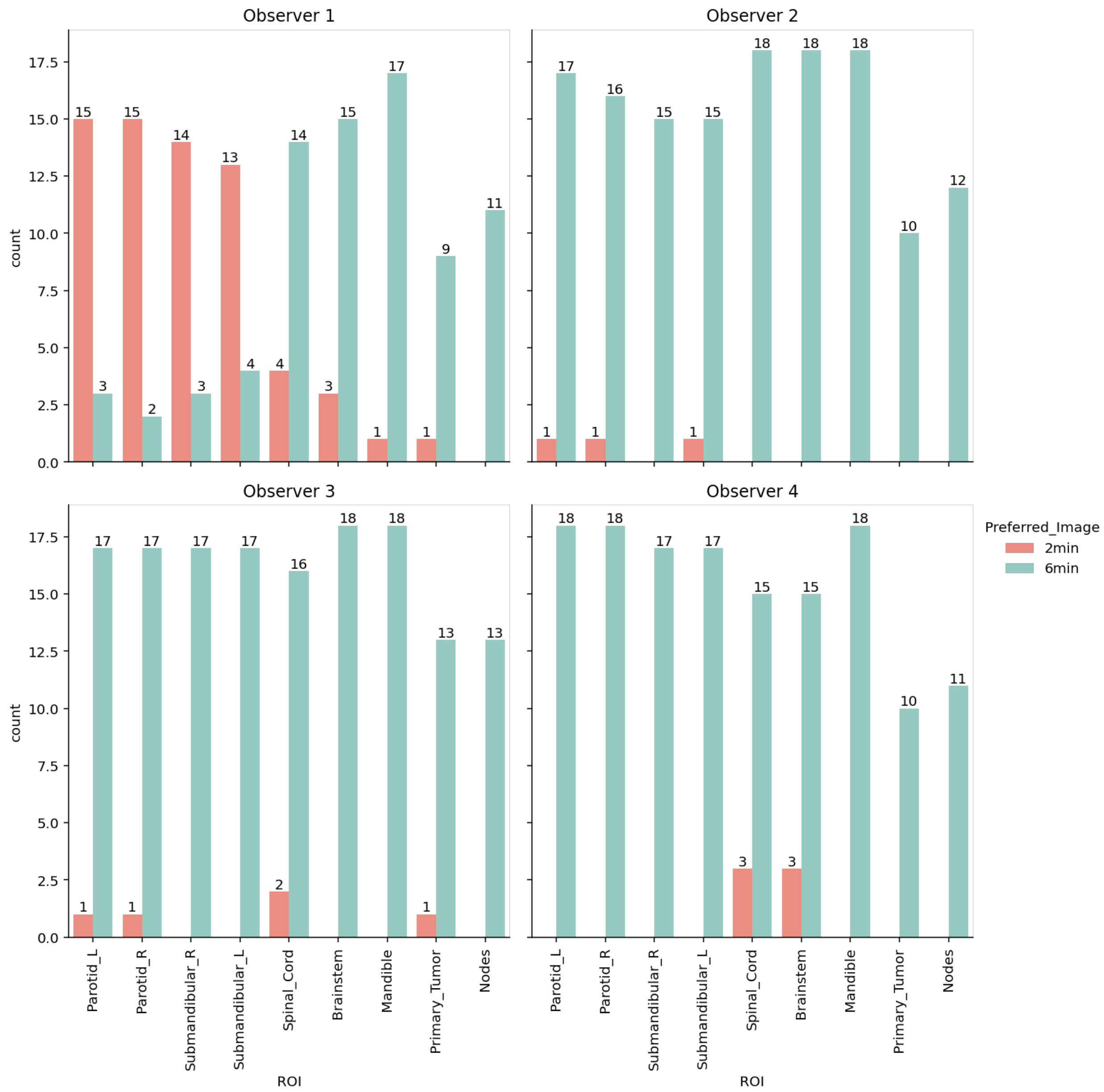

Figure C1. Observer preferences for visualizing regions of interest (ROI) on ground-truth 2-minute scans (red) vs. ground-truth 6-minute scans (green) for a variety of ROIs.

Appendix D. Additional Auto-segmentation Data

A previously trained head and neck cancer organ at risk (OAR) auto-segmentation model initially developed in independent 2-minute MRI scans was applied to the ground-truth 2-minute, ground-truth 6-minute, and synthetic 6-minute MRI scans in the test set. Examples of auto-segmented OARs overlaid on images and in 3D volumetric format for ground-truth 2-minute and ground-truth 6-minute scans in one case are shown in **Figure D1**.

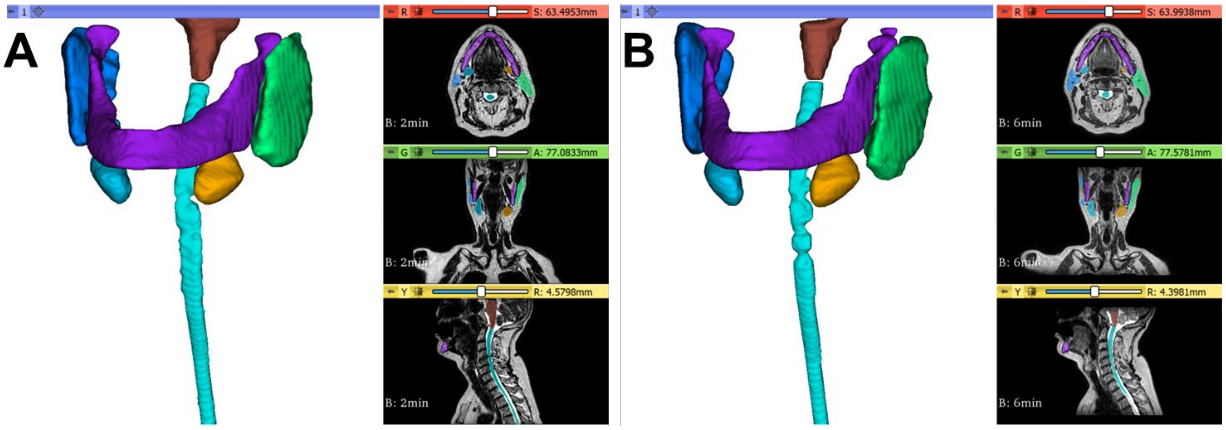

Figure D1. Organ at risk auto-segmentation 3D representation and axial/coronal/sagittal views for ground-truth 2-minute **(A)** and ground-truth 6-minute **(B)** scans for 1 representative case where all structures were correctly contoured. Right parotid gland, left parotid gland, left submandibular gland, right submandibular gland, spinal cord, brainstem, and mandible, are represented by the dark blue, green, light blue, orange, teal, brown, and purple structures. Visualizations generated in 3D Slicer.

Interobserver variability (IOV) cutoffs for each OAR were determined for the Dice similarity coefficient (DSC) and average surface distance (ASD) from supporting data in work by McDonald et al. (10). For equivalence tests, we also implemented the interquartile range (IQR) values as the minimum (-IQR) and maximum (+IQR) equivalence bounds. A table of the estimated values is shown below (**Table D1**).

Table D1. Median (med) and interquartile range (IQR) values for expert interobserver Dice similarity coefficient (DSC) and average surface distance (ASD) for each organ at risk structure.

Structure	Med DSC	IQR DSC	Med ASD	IQR ASD
Left Parotid Gland	0.83	0.08	1.80	1.05
Right Parotid Gland	0.84	0.07	1.50	1.00
Mandible	0.71	0.10	1.40	1.10

Spinal Cord	0.85	0.10	0.60	0.20
Brainstem	0.86	0.07	1.30	0.40
Left Submandibular Gland	0.75	0.12	1.40	1.20
Right Submandibular Gland	0.78	0.11	1.40	1.00

In the main manuscript we do not include the OARs whose median values between ground-truth 6-minute and ground-truth 2-minute scans do not cross the corresponding IOV median value (i.e., lower than threshold in case of DSC or higher than threshold in case of ASD) as these structures would likely not be clinically acceptable, i.e., spinal cord, brainstem, left/right submandibular glands. However, for completeness, we show the full bar plot representations for all OARs below (**Figure D2**). DSC and ASD equivalence tests (two one-sided t-tests) were non-significant ($p > 0.05$) for the spinal cord, brainstem, left submandibular gland, and right submandibular gland. Finally, we also show DSC and ASD bar plots for all OARs for direct comparisons of ground-truth to synthetic 6-minute images in **Figure D3**.

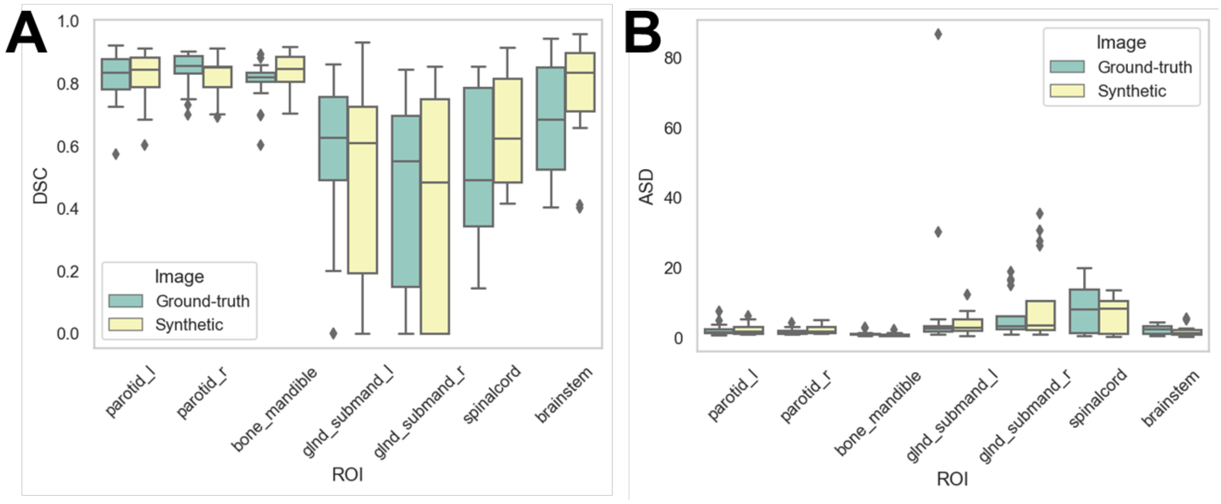

Figure D2. Complete auto-segmentation results for image (ground-truth or synthetic) vs. 2-minute scan. Auto-segmented organs at risk were generated on ground-truth (green) or synthetic (yellow) 6-minute images and compared against ground-truth 2-minute images using the **(A)** Dice similarity coefficient (DSC) and **(B)** average surface distance (ASD).

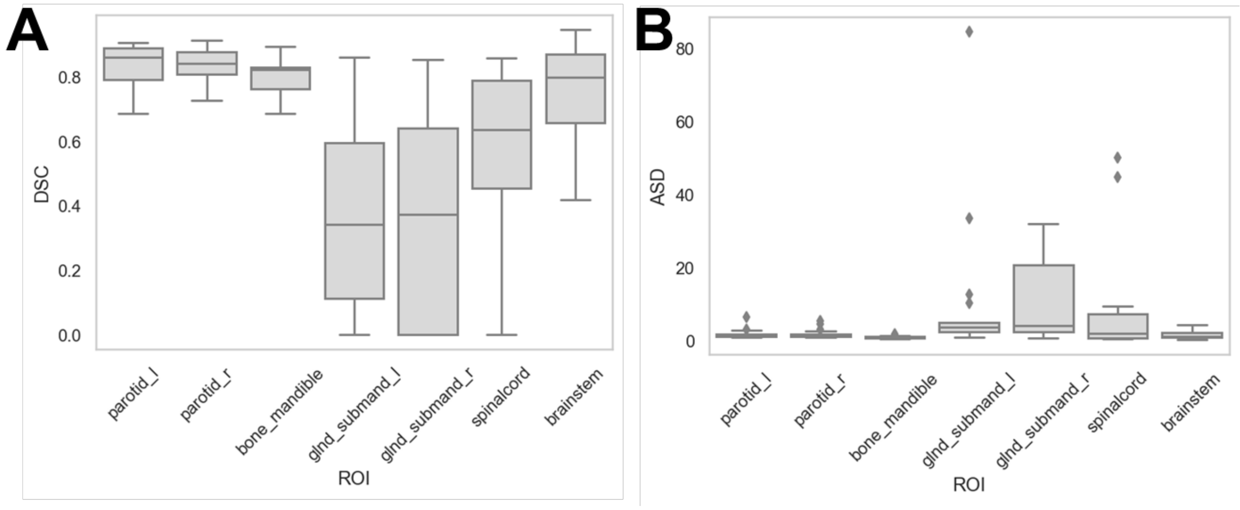

Figure D3. Complete auto-segmentation results for ground-truth vs. synthetic 6-minute scans. **(A)** Dice similarity coefficient (DSC) and **(B)** average surface distance (ASD).

Appendix E. Additional Image Similarity Data

In **Table E1** we show image similarity metrics (mean squared error [MSE], structural similarity index [SSIM], and peak signal to noise ratio [PSNR]) for the deep learning model without N4 bias field correction, with N4 bias field correction before application of a sharpening kernel (main results described in manuscript), and with N4 bias field correction after application of a sharpening kernel. Generally, metrics improved slightly or remained similar with N4 bias field correction, and worsened after application of the sharpening kernel.

Table E1. Image similarity results across the whole image and various subregions for the deep learning model without N4 bias field correction, with N4 bias field correction, and with N4 bias field correction after application of a sharpening kernel. Values presented are rounded up to two decimal places. MSE = mean squared error, SSIM = structural similarity index, PSNR = peak signal to noise ratio.

ROI	Model	MSE	SSIM	PSNR
Whole	without N4	0.19 (0.06)	0.93 (0.03)	33.41 (2.13)
Whole	with N4	0.19 (0.05)	0.93 (0.03)	33.14 (2.30)
Whole	with N4 sharpened	0.22 (0.06)	0.92 (0.04)	32.27 (2.17)
Mandible	without N4	1.20 (0.71)	0.56 (0.08)	23.21 (2.41)
Mandible	with N4	1.16 (0.50)	0.56 (0.09)	23.33 (1.83)
Mandible	with N4 sharpened	1.47 (0.64)	0.51 (0.08)	22.16 (1.84)
Brainstem	without N4	5.28 (3.13)	0.67 (0.10)	19.37 (2.11)
Brainstem	with N4	4.99 (2.98)	0.66 (0.09)	19.17 (2.03)
Brainstem	with N4 sharpened	6.45 (3.21)	0.61 (0.09)	18.07 (1.72)
External	without N4	0.43 (0.15)	0.80 (0.05)	30.09 (1.29)
External	with N4	0.41 (0.12)	0.80 (0.06)	30.06 (1.32)

External	with N4 sharpened	0.51 (0.14)	0.79 (0.07)	28.96 (1.33)
Left Submandibular Gland	without N4	1.77 (1.38)	0.40 (0.08)	18.82 (3.30)
Left Submandibular Gland	with N4	1.42 (0.79)	0.43 (0.12)	19.52 (3.56)
Left Submandibular Gland	with N4 sharpened	1.95 (0.98)	0.39 (0.14)	18.12 (3.67)
Right Submandibular Gland	without N4	1.71 (1.43)	0.48 (0.16)	17.73 (3.83)
Right Submandibular Gland	with N4	1.44 (0.84)	0.48 (0.14)	18.02 (3.34)
Right Submandibular Gland	with N4 sharpened	1.95 (1.21)	0.43 (0.15)	16.90 (3.27)
Left Parotid Gland	without N4	1.40 (0.72)	0.47 (0.10)	19.39 (3.54)
Left Parotid Gland	with N4	1.40 (0.52)	0.46 (0.11)	18.87 (2.64)
Left Parotid Gland	with N4 sharpened	1.92 (0.64)	0.41 (0.12)	17.57 (2.45)
Right Parotid Gland	without N4	1.32 (0.98)	0.53 (0.09)	19.58 (2.21)
Right Parotid Gland	with N4	1.20 (0.52)	0.52 (0.11)	19.38 (1.95)
Right Parotid Gland	with N4 sharpened	1.63 (0.67)	0.48 (0.11)	18.18 (1.67)
Spinal Cord	without N4	2.01 (2.19)	0.51 (0.09)	22.36 (2.49)
Spinal Cord	with N4	1.98 (2.04)	0.51 (0.09)	22.56 (2.45)
Spinal Cord	with N4 sharpened	2.83 (2.53)	0.45 (0.09)	21.09 (2.11)

In addition to similarity metric analysis, we also performed a preliminary investigation of radiomic features for synthetic vs. ground-truth images. Specifically, we sought to determine the reliability/repeatability of various radiomic feature classes for the previously segmented OARs on the synthetic images generated by the model described in the main manuscript (with N4 bias

field correction before application of sharpening kernel). OAR segmentations on 2-minute scans were resampled to the ground-truth 6-minute and synthetic 6-minute scans using a nearest neighbor interpolator. As before, auto-segmented structures for patients which were not present (i.e., glands post-resection) were not included in the analysis. Radiomic feature extractions were performed on z-score normalized images. Using the open-source toolkit, PyRadiomics (11), we extracted the standard default features from first order statistics (firstorder; 19 features), grey level co-occurrence matrix (glcm; 24 features) gray level run length matrix (glrlm; 16 features), neighbouring gray tone difference matrix (ngtdm; 5 features), and gray level dependence matrix (gldm; 14 features) from OARs on ground-truth 6-minute and synthetic 6-minute scans. The default PyRadiomics extraction parameters, e.g., fixed bin width, were applied as recommended. Shape features were not extracted since the OAR mask was the same between the scans. We utilized the two-way mixed effects, consistency, single rater/measurement intraclass correlation coefficient (ICC) provided by the pinguoin Python package (12) to calculate ICC values for each feature class/OAR combination. ICC targets were individual patients, raters were the different images (ground-truth, synthetic), and ratings were the OAR radiomic feature values. ICC values less than 0.5 were categorized as non-reliable, while ICC values greater than or equal to 0.5 were categorized as reliable. The ICC results stratified by OAR and radiomic feature category are shown in **Figure E1**. Generally, a greater number of firstorder features were considered reproducible than non-reproducible for most OARs. For gldm and glrlm, a smaller proportion of features were considered reproducible for most OARs. For ngtdm, only a small number of features for the spinal cord and brainstem were considered reproducible. Finally, for glcm and glszm no features were considered reproducible. Future work should investigate the utility of using synthetic images for radiomic feature calculation in MRI-guided adaptive radiotherapy workflows in greater depth.

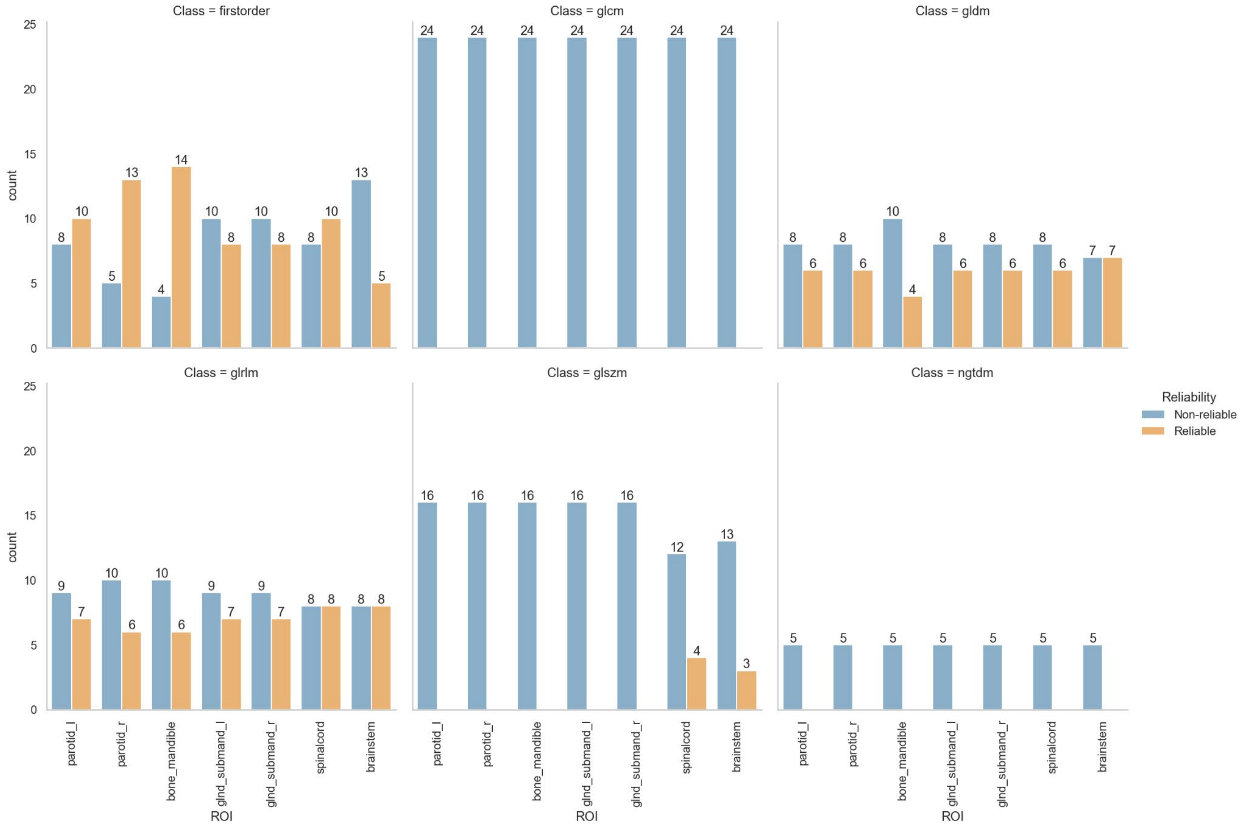

Figure E1. Radiomic feature reliability/repeatability on synthetic scans compared to ground-truth scans stratified by region of interest (ROI) and feature category. firstorder = first order statistics, glcm = grey level co-occurrence matrix, glrlm = gray level run length matrix, ngtdm = neighbouring gray tone difference matrix, gldm = gray level dependence matrix.

Appendix F. Turing Test Additional Data

The Turing test was initially performed with raw image outputs and subsequently after the application of a simple sharpening kernel. The same expert physician observers were given re-randomized slice representations of the same cases one week after the initial Turing test. Only the results after application of the sharpening kernel are displayed in the main manuscript. For completeness we display the results of the Turing test without the sharpening kernel below. As opposed to the results with the application of the sharpening kernel, the original outputs were often distinguishable due to a slight systematic blurring effect. **Table F1** shows the Turing test and clinician preference results while **Figure F1** shows the stratified clinician preference results.

Table F1. Turing test and image preference results for three physician expert observers before application of sharpening kernel. Each observer was asked to determine the image identity of blinded paired ground truth (GT) or synthetic 6-minute MRI scan slices in a randomized fashion and also provide their preference. Two one sided tests for two proportions were applied to determine if observer estimates were equivalent to chance.

Observer	% Correct	p-val	% GT Preference	p-val
1	98	0.99999997	93	0.999979105
2	68	0.38496071	62	0.125479502
3	65	0.23466347	58	0.043816053

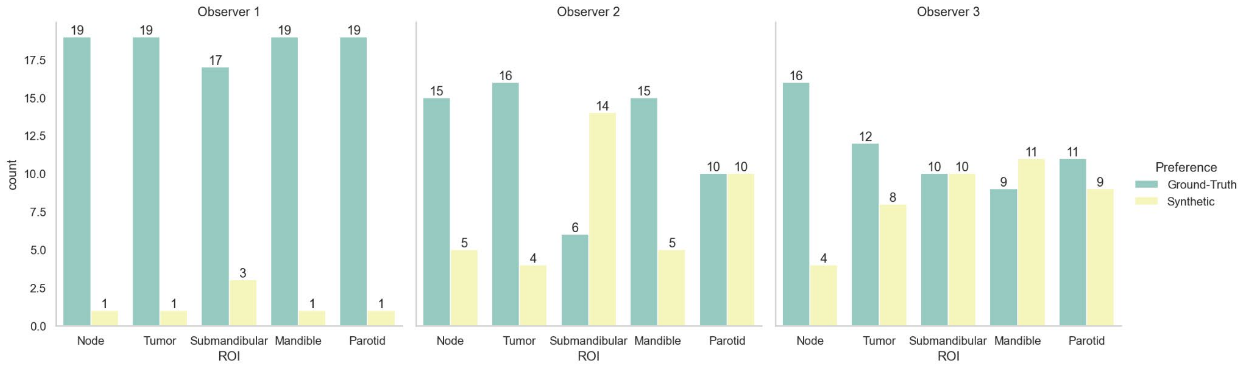

Figure F1. Clinician image preferences stratified by region for Turing test before application of sharpening kernel. Green = ground-truth 6-minute MRI slice, yellow = synthetic 6-minute MRI slice.

Additionally, observers were instructed to provide comments where desired to indicate specific reasons for categorizing images as either ground-truth or synthetic. The raw comments for the

Turing test before (**Table F2**) and after (**Table F3**) application of the sharpening kernel are shown below for each observer.

Table F2. Turing test comments for each observer before application of sharpening kernel.

Observer	Slice ID	Left image impression	Right image impression	I prefer	Comments
1	1	AI	Real	Left	Left has better margins between structures
1	2	Real	AI	Right	Right has better resolution
1	5	Real	AI	Right	Right has better visualization of tissues
1	6	AI	Real	Left	Left has better resolution
1	7	AI	Real	Left	Left has less noise
1	9	AI	Real	Left	Left has better contrast between tumor and surrounding normal tissues
1	12	Real	AI	Right	Right shows better visualization of tumor
1	15	Real	AI	Right	There is better discrimination of tumor in the rigth
1	17	Real	AI	Right	Right shows better discrimination between tumor and submandibular gland
1	18	Real	AI	Right	Right has better visualization of LN
1	20	AI	Real	Left	Left has better margins between tissues
1	21	Real	AI	Right	Right has better discrimination between LN and Rt submandibular gland
1	22	AI	Real	Left	Left has better visualization of tumor
1	25	AI	Real	Left	Left has better quality in defining Lt RP LN
1	26	AI	Real	Left	Left has better visualization of parotids

1	27	Real	AI	Right	Right has better visualization of Rt level IB LN
1	28	Real	AI	Right	Right has better discrimination of Rt cervical LN
1	31	AI	Real	Left	Left has better visualization of tumor
1	32	AI	Real	Left	Left has better visualization of tumor
1	34	AI	Real	Left	Left has better visualization of LN margins
1	36	AI	Real	Left	Left has better visualization of LN margins
1	38	Real	AI	Right	Right has better visualization of normal tissues' margins
1	39	AI	Real	Left	Left has better visualization of normal tissues' margins
1	41	AI	Real	Left	Left has better visualization of tumor
1	44	AI	Real	Left	Left has better discrimination of Lt RP LN
1	45	AI	Real	Left	Left has better visualization of LN margins
1	48	AI	Real	Left	Left has better demarcation of normal tissues
1	53	AI	Real	Left	Left has better descrimination of primary tumor
1	54	Real	AI	Right	Right has better discrimination of primary and nodal volumes
1	57	Real	AI	Right	Right has better demarcation of LN
1	59	AI	Real	Left	Left has better discrimination of tumor
1	63	AI	Real	Left	Left has better visualization of GTVP, GTVN
1	64	AI	Real	Left	Left has better defining og LN margins

1	65	AI	Real	Left	Left has better discrimination between Lt submandibular gland and LN
1	66	AI	Real	Left	Left has better visualization of Lt RP LN
1	71	Real	AI	Right	LN can be better visualized at right image
1	75	AI	Real	Right	Right has better discrimination of primary and nodal volumes
1	79	Real	AI	Right	Right has better visualization of Rt LN
1	80	Real	AI	Right	Right has better discrimination between LN and Lt submandibular gland
1	83	AI	Real	Left	Left has better discrimination of Lt primary tumor
1	84	AI	Real	Right	Right has better visualization of normal structures
1	86	Real	AI	Right	Better visualization of tumor at right image
1	88	Real	AI	Right	Better detection of Lt RP LN at right image
1	89	Real	AI	Right	Better detection of GTVP, GTVN at right image
1	93	Real	AI	Right	Better discrimination between LN and Rt submandibular gland at right image
1	94	Real	AI	Right	Can't actually see great difference between both
1	95	Real	AI	Left	Can see more noise at Rt image
1	100	Real	AI	Right	Better visualization of LN at right image
2	1	AI	Real	Left	Left has better margins between structures
2	3	Real	AI	Left	Submandibular gland on Rt looks calccified when it might not
2	4	AI	Real	Right	sublingual glands aren't visible on Lt

2	8	Real	AI	Right	less noise
2	16	Real	AI	Left	sublingual glands not clear
3	1	AI	Real	Right	better details
3	6	AI	Real	Right	Rt vascular vessles seen better
3	7	AI	Real	Right	Rt tumor boundries seen better
3	8	AI	Real	Right	Rt vascular space more detailed
3	9	AI	Real	Left	better LN margins
3	10	AI	Real	Right	vessles better
3	11	Real	AI	Right	tongue mass better delination
3	15	AI	Real	Right	part of Lt vertabrae not clear
3	44	Real	AI	Left	part of image is missing
3	46	AI	Real	Right	hazy vasless and pharynex
3	62	AI	Real	Right	pharyngeal constrictor and vessles betterseen
3	66	AI	Real	Left	better parotid
3	68	Real	AI	Left	tumor better seen
3	75	Real	Real	Right	gross tumor better seen

Table F3. Turing test comments for each observer after application of sharpening kernel.

Observer	Slice _ID	Left image impression	Right image impression	I prefer	Comments
1	1	AI	Real	Left	Left has better margins between structures
1	2	AI	Real	Left	Better visualization of Rt level 2 cervical LN
1	3	Real	AI	Right	Better visualization of tumor at BOT
1	6	AI	Real	Right	Better visualization of submandibular glands at Rt
1	7	Real	AI	Left	Better demarcation between sbmandibular gland and cervical LN at LT
1	8	Real	AI	Right	Better visualization of Level 2 cervical LN at Rt
1	9	Real	AI	Right	GTV P & N are better seen at Rt
1	12	Real	AI	Right	Better visualization of normal tissues at Rt
1	14	AI	Real	Left	GTVP is better seen at L
1	15	AI	Real	Right	Less noise at Rt
1	17	AI	Real	Right	Better visualization of tumor at Rt
1	19	AI	Real	Left	Level 2 LN is better seen at Lt
1	20	Real	AI	Right	Less noise at Rt
1	22	AI	Real	Left	Better demarcation of submandibular glands at Lt
1	23	AI	Real	Right	Level 2 cervical LN appears cystic at Lt, while it may be not
1	24	AI	Real	Right	Better visulaization of GTVP at Rt
1	25	Real	AI	Left	Better visulaization of GTVP at Lt

1	28	Real	AI	Right	Lt RP is better seen at Rt
1	29	AI	Real	Left	GTVP, GTVN are better seen at Lt
1	30	Real	AI	Left	GTVP, GTVN are better seen at Lt
1	35	Real	AI	Right	Rt has less noise, better visulaization of tumor
1	41	Real	AI	Right	Lt RP is better seen at Rt
1	43	AI	Real	Left	Better visualization of GTVP, GTVN at Lt
1	44	AI	Real	Right	GTVP is better seen at Rt
1	45	AI	Real	Left	GTVN is better seen at Lt
1	47	Real	AI	Left	Tumor is better seen at Lt
1	48	AI	Real	Left	Level 2 cervical LN is better seen at Lt
1	50	AI	Real	Left	GTVP is better seen at Lt
1	52	Real	AI	Left	Less noise at Lt
1	55	AI	Real	Right	Better visualization of tumor
1	56	AI	Real	Left	Rt level 2 cervical LN is better seen at Lt
1	59	AI	Real	Left	Less noise at Lt
1	61	AI	Real	Left	Lt RP is better seen at Lt
1	68	Real	AI	Left	Tumor is better seen at Lt
1	69	AI	Real	Left	Less noise at Lt

1	70	AI	Real	Right	Tumor is better seen at Rt
1	73	AI	Real	Left	Better demarcation of tissues at Lt
1	76	Real	AI	Right	Tumor is better seen at Rt
1	79	Real	AI	Right	Better denarcation of cervical LN at Rt
1	80	AI	Real	Right	Less noise at Rt
1	88	AI	Real	Left	Better denmarcation of tissues, less noise at Lt
1	90	Real	AI	Left	GTVP is better seen at Lt
1	91	Real	AI	Right	Better demarcation between sbmandibular gland and cervical LN at LT
1	94	Real	AI	Right	Better demarcation of cervical LN at Rt
1	96	Real	AI	Right	Better demarcation of normal tissues
1	99	Real	AI	Right	GTVP is better seen at Rt
1	100	Real	AI	Right	Lt RP is better seen at Rt
3	1	Real	AI	Left	vessles more clear
3	2	AI	Real	Right	artificate on the Rt II
3	4	AI	Real	Right	larynex more clear
3	10	AI	Real	Right	both vertabre not seen
3	22	Real	AI	Left	subman gland more evident
3	26	Real	AI	Left	both tongue is heterogenous

3	27	AI	Real	Right	vertabrae and muscle are bad both
3	36	AI	Real	Right	vertabrae and muscle are bad both
3	40	AI	Real	Right	vertabrae and muscle are bad both
3	54	Real	AI	Left	not clear details
3	65	AI	Real	Right	disturbed anatomy both in muscles and vetrtabrae
3	72	AI	Real	Left	anatomy not clear vertabrae and muscles
3	84	AI	Real	Right	junction between larynex and vertabrae poorly seen
3	93	AI	Real	Right	disturbed arrangement
3	98	AI	Real	Right	pharnex not clear
3	99	Real	AI	Left	muscles spaces and glands are not clearly lined

References

1. Goodfellow I, Pouget-Abadie J, Mirza M, Xu B, Warde-Farley D, Ozair S, Courville A, Bengio Y. Generative Adversarial Nets. *Adv Neural Inf Process Syst* (2014) 27: https://proceedings.neurips.cc/paper/5423-generative-adversarial-nets [Accessed May 5, 2022]

2. Zhu J-Y, Park T, Isola P, Efros AA. Unpaired image-to-image translation using cycle-consistent adversarial networks. *Proceedings of the IEEE international conference on computer vision*. (2017). p. 2223–2232

3. Abadi M, Agarwal A, Barham P, Brevdo E, Chen Z, Citro C, Corrado GS, Davis A, Dean J, Devin M, et al. TensorFlow: Large-Scale Machine Learning on Heterogeneous Distributed Systems. *arXiv [csDC]* (2016) http://arxiv.org/abs/1603.04467

4. Johnson J, Alahi A, Fei-Fei L. Perceptual Losses for Real-Time Style Transfer and Super-Resolution. *Computer Vision – ECCV 2016*. Springer International Publishing (2016). p. 694–711

5. Isola P, Zhu J-Y, Zhou T, Efros AA. Image-to-image translation with conditional adversarial networks. *Proceedings of the IEEE conference on computer vision and pattern recognition*. (2017). p. 1125–1134

6. Li C, Wand M. Precomputed Real-Time Texture Synthesis with Markovian Generative Adversarial Networks. *Computer Vision – ECCV 2016*. Springer International Publishing (2016). p. 702–716

7. Ledig C, Theis L, Huszár F, Caballero J, Cunningham A, Acosta A, Aitken A, Tejani A, Totz J, Wang Z, et al. Photo-realistic single image super-resolution using a generative adversarial network. *Proceedings of the IEEE conference on computer vision and pattern recognition*. (2017). p. 4681–4690

8. Maes F, Collignon A, Vandermeulen D, Marchal G, Suetens P. Multimodality image registration by maximization of mutual information. *IEEE Trans Med Imaging* (1997) 16:187–198.

9. Pieper S, Halle M, Kikinis R. 3D Slicer. *2004 2nd IEEE International Symposium on Biomedical Imaging: Nano to Macro (IEEE Cat No. 04EX821)*. (2004). p. 632-635 Vol. 1

10. McDonald BA, Cardenas C, O'Connell N, Ahmed S, Naser MA, Wahid KA, Xu J, Thill D, Zuhour R, Mesko S, et al. Investigation of autosegmentation techniques on T2-weighted MRI for off-line dose reconstruction in MR-linac Adapt to Position workflow for head and neck cancers. *bioRxiv* (2021) doi: 10.1101/2021.09.30.21264327

11. van Griethuysen JJM, Fedorov A, Parmar C, Hosny A, Aucoin N, Narayan V, Beets-Tan RGH, Fillion-Robin J-C, Pieper S, Aerts HJWL. Computational Radiomics System to Decode the Radiographic Phenotype. *Cancer Res* (2017) 77:e104–e107.

12. Vallat R. Pingouin: statistics in Python. *J Open Source Softw* (2018) 3:1026.
